# EIF3M as a pan-cancer biomarker: prognostic significance and immune infiltration association

**DOI:** 10.3389/fmolb.2025.1697083

**Published:** 2025-11-18

**Authors:** Zhilei Zhao, Jiaxing Chen, Yongqiang Cui, Zhihao Fu, Dongfeng Deng, Xiao Zhang

**Affiliations:** 1 Zhengzhou University People’s Hospital, Henan Provincial People’s Hospital, Zhengzhou, Henan, China; 2 Department of Hepatobiliary Pancreatic Surgery, Henan Provincial People’s Hospital, Zhengzhou, Henan, China; 3 Department of Hepatobiliary Pancreatic Surgery, Henan Provincial People’s Hospital, Zhengzhou University People’s Hospital and Henan University People’s Hospital, Zhengzhou, Henan, China

**Keywords:** EIF3M, pan-cancer, biomarkers, immune microenvironment, prognosis

## Abstract

**Background:**

EIF3M, a core subunit of eukaryotic translation initiation factor 3, plays a pivotal role in protein synthesis by regulating the assembly of the 43S initiation complex. However, its biological functions in cancer remain poorly understood. To further investigate the clinical translational value and underlying mechanisms of EIF3M in tumors, this study conducted comprehensive bioinformatic analysis of EIF3M across various tumor types.

**Methods:**

We utilized publicly available databases to perform a comprehensive bioinformatics analysis of EIF3M’s biological roles in oncogenesis, aiming to elucidate its pan-cancer expression patterns and prognostic significance. Furthermore, we conducted an integrative multi-omics analysis incorporating methylation profiling, co-expressed gene networks, targeted miRNA interactions, and tumor immune microenvironment infiltration to decipher the complex regulatory architecture and biological pathways mediated by EIF3M across cancer types. Finally, we used HCC cell lines for *in vitro* functional validation, determining how EIF3M expression modulates malignant phenotypic behaviors in hepatocellular carcinoma.

**Results:**

EIF3M was overexpressed in multiple cancers and correlated with advanced tumor stage and poor survival. Its dysregulation was primarily driven by gene amplification and regulated by promoter methylation and miRNAs. EIF3M functioned as a hub in cell cycle and transcriptional networks and was linked to an immunosuppressive microenvironment. In hepatocellular carcinoma models, EIF3M modulated tumor proliferation, migration, and activated oncogenic pathways like Wnt/β-catenin.

**Conclusion:**

This study reveals that EIF3M expression correlates with immune infiltration and poor prognosis in multiple cancers. In vitro experiments in hepatocellular carcinoma models demonstrated that *EIF3M* critically regulates malignant cell behaviors. Collectively, our findings highlight EIF3M’s value as a promising pan-cancer biomarker worthy of further investigation for its utility in prognosis prediction and as an indicator of immunotherapeutic response.

## 1 Introduction

Malignant tumors have emerged as a major challenge in the global public health arena. In most high-income countries, they have risen to the top cause of death among residents. Meanwhile, in middle- and low-income countries, both the incidence and mortality rates of malignant tumors are showing a notable upward trend (Chen et al., 2025). According to statistical reports, there were 19.3 million newly diagnosed malignant tumor cases and approximately 10 million malignant tumor-related deaths globally in 2020. The report also projects that the global burden of malignant tumors will rise to 28.4 million cases by 2040, representing a 47% increase from 2020 (Siegel et al., 2024). Despite significant improvements in the five-year survival rates of malignant tumor patients over recent decades, a substantial proportion of these individuals continue to face persistently poor survival outcomes, presenting an ongoing clinical challenge. For instance, prognosis remains particularly poor for patients with certain tumors such as pancreatic cancer (Maomao et al., 2022). Concurrently, current cancer therapeutics continue to face significant challenges in efficacy. Despite the adoption of multimodal treatment strategies—primarily based on surgical resection combined with radiotherapy and chemotherapy—patients frequently encounter issues such as incomplete tumor removal, postoperative recurrence, and chemotherapy resistance, leading to generally poor prognoses. The biological complexity of these malignancies, including high invasiveness, an immunosuppressive microenvironment, and activation of multiple drug-resistance mechanisms, substantially limits the clinical benefits of existing therapies. Consequently, the development of novel targeted strategies capable of overcoming therapeutic resistance—particularly that driven by the dynamic evolution of the tumor microenvironment—remains a critical and ongoing focus in oncology research (Siegel et al., 2024; Sung et al., 2021; Guo et al., 2022; Yang et al., 2024).

Eukaryotic translation initiation factor 3 subunit M (EIF3M) functions as a central regulatory molecule in protein biosynthesis, with its encoded product forming a core structural component of the eukaryotic translation initiation factor 3 (EIF3) complex. Extensive research has established that this complex operates as a pivotal regulatory hub governing critical processes including translation initiation, termination, and ribosome recycling, thereby serving as an indispensable molecular foundation for the regulatory network controlling eukaryotic protein synthesis (Gomes-Duarte et al., 2018). In recent years, research evidence on EIF3M in the field of oncology has been progressively accumulating, revealing its potential regulatory role in tumorigenesis and progression. Studies have reported that silencing *EIF3M* expression in colorectal cancer cell lines significantly suppressed the malignant biological behaviors of these cells (Goh et al., 2011). Similar investigations have also revealed that *EIF3M* exhibits overexpression in prostate cancer and triple-negative breast cancer, with cellular experiments confirming significant growth inhibition in tumor cells following *EIF3M* knockdown (Guo et al., 2024; Han et al., 2020). In lung cancer research, investigators have identified that *EIF3M* interacts with genes including SAAL1 and CAPRIN1 to promote tumorigenesis and cancer progression (Chiang et al., 2024; Liu et al., 2021). Although existing research has begun to elucidate the expression profiles and molecular regulatory mechanisms of *EIF3M* in specific cancer types, a systematic understanding of its pan-cancer expression patterns, comprehensive biological functional landscape, and regulatory characteristics within signaling networks remains largely unexplored. Consequently, our research group aims to transcend the traditional cancer classification based on tissues or organs, and instead investigate the commonalities and heterogeneities of *EIF3M* across multiple cancer types from molecular and genomic perspectives.

This study aims to systematically dissect the multidimensional molecular characteristics and clinical significance of EIF3M in pan-cancer contexts. By integrating multi-omics data derived from multiple public databases, we seek to elucidate its aberrant overexpression across multiple malignancies and its significant association with poor patient prognosis. Furthermore, we investigated the correlations between *EIF3M* mutations, epigenetic regulation, and tumorigenesis, while revealing the underlying mechanisms of cancer progression mediated through regulatory networks driven by interacting genes and miRNAs. Additionally, we explored the relationship between *EIF3M* expression levels and remodeling of the tumor microenvironment. This research not only expands theoretical understanding of EIF3M’s role in cancer heterogeneity regulation but also provides critical molecular evidence and potential therapeutic targets for developing EIF3M-based prognostic evaluation systems, targeted therapies, and immuno-combination therapeutic strategies.

## 2 Materials and methods

### 2.1 Data collection and analysis

The Tumor Immune Estimation Resource 2.0 (TIMER2.0, http://timer.cistrome.org) resource repository integrates multi-omics data from multiple large-scale cancer genomic cohorts and other public databases, facilitating comprehensive insights into the expression profiles of *EIF3M* across pan-cancer contexts (Li et al., 2020). Gene Expression Profiling Interactive Analysis 2 (GEPIA2, http://gepia2.cancer-pku.cn)specializes in gene expression profiling studies across cancerous and normal tissues (Tang et al., 2019). By integrating large-scale RNA sequencing datasets from The Cancer Genome Atlas (TCGA) and the Genotype-Tissue Expression (GTEx) projects, this platform facilitates comprehensive investigation of *EIF3M* expression disparities between multiple cancer types and their matched normal tissues, as well as its associations with clinical characteristics and survival curve differentiations (De Mendonça et al., 2025; Pastor and Hong, 2023). The University of Alabama at Birmingham Cancer Data Analysis Portal database (UALCAN, https://ualcan.path.uab.edu) is a web-based platform dedicated to mining cancer multi-omics data and investigating clinical correlations, capable of providing protein expression profiles of *EIF3M* through integration with the Clinical Proteomic Tumor Analysis Consortium (CPTAC) database (Chandrashekar et al., 2022; Prakash et al., 2021). The Human Protein Atlas (HPA, https://www.proteinatlas.org) integrates antibody-based proteomics data, RNA sequencing data, and pathological imaging to systematically characterize the expression and localization patterns of EIF3M in normal tissues (Gu et al., 2025). This platform further provides comparative immunohistochemical (IHC) images of EIF3M expression in paired normal and tumor tissues. All aforementioned statistical analyses were conducted through automated computational pipelines integrated within online platforms. Additionally, we retrieved *EIF3M* expression data from the TCGA database across multiple cancer types, and utilized these datasets as the foundation to conduct disease-specific survival (DSS) and progression-free survival (PFS) curve analyses, as well as to calculate gene activity scores for *EIF3M*.

### 2.2 Integrative analysis of EIF3M mutations and methylation

The cBio Cancer Genomics Portal (cBioPortal, https://www.cbioportal.org) integrates multi-omics data, including somatic mutations, copy number alterations (CNAs), expression profiles and clinical information, enabling comprehensive analysis of *EIF3M* mutation frequencies, CNAs, and other genomic alterations across cancer contexts (De Bruijn et al., 2023). Furthermore, we calculated the tumor mutational burden (TMB) of *EIF3M* across multiple cancer types using data from TCGA database (Endris et al., 2019). The Shiny Methylation Analysis Resource Tool (SMART) integrates DNA methylation data from 33 malignancies within TCGA database (Li et al., 2019). Its functionalities span single-locus CpG site analysis to pan-cancer methylation landscape profiling, enabling systematic investigation of CpG site methylation levels for *EIF3M*. The UALCAN database provides foundational data for analyzing *EIF3M* promoter methylation, integrating multi-omics datasets to explore epigenetic regulation in cancer contexts (Chandrashekar et al., 2022).

### 2.3 Functional and pathway enrichment analysis based on related genes

Pathway Commons (https://www.pathwaycommons.org) is an integrated biological pathway database that aggregates pathway information from multiple sources, including KEGG, Reactome, BioCyc, and WikiPathways (Cerami et al., 2011). This platform enables the identification of gene sets exhibiting strong associations with *EIF3M* and further facilitates the analysis of differential expression levels of these gene sets across pan-cancer contexts. Enrichment Analysis (Enrichr, https://maayanlab.cloud/Enrichr) serves as an online platform dedicated to functional annotation and enrichment analysis of gene sets, integrating over 200 functional annotation databases (Subramanian et al., 2005). It stands as a cornerstone tool in functional genomics research, enabling the exploration of highly enriched ontological features and signaling pathways associated with gene sets linked to EIF3M. The Search Tool for the Retrieval of Interacting Genes/Proteins (STRING, https://cn.string-db.org) is a public database dedicated to predicting protein-protein interactions (PPIs) and analyzing functional associations (Szklarc et al., 2023). This platform enables the identification of proteins potentially interacting with EIF3M and elucidates their involvement in molecular pathways.

### 2.4 Bioinformatic analysis of predicted miRNAs

miRDB (https://mirdb.org), TargetScan (https://www.targetscan.org), and miRWalk (http://mirwalk.umm.uni-heidelberg.de) are three widely utilized databases in bioinformatics, all specializing in microRNA (miRNA) target prediction and functional analysis (Wong and Wang, 2015; Agarwal et al., 2015; Dweep et al., 2011). By intersecting results from these databases, highly credible target miRNAs and their binding sites for *EIF3M* can be predicted. The Database of Differentially Expressed MiRNAs in Human Cancers (dbDEMC, https://www.biosino.org/dbDEMC) is a public repository specifically focused on cataloging differentially expressed microRNAs (miRNAs) in cancer (GPB, 2024). It integrates miRNA expression data derived from high-throughput studies, providing a platform for systematic investigation of the expression profiles of *EIF3M*-targeting miRNAs across pan-cancer contexts. The Encyclopedia of RNA Interactomes (ENCORI, also known as starBase, https://rnasysu.com/encori/) is a specialized platform for investigating regulatory interactions between non-coding RNAs and coding RNAs or proteins (Li et al., 2014). It facilitates systematic exploration of *EIF3M*-targeting miRNAs, enabling pan-cancer correlation analyses between these miRNAs and *EIF3M*, as well as functional enrichment assessments to identify associated biological pathways or molecular mechanisms.

### 2.5 Pan-cancer investigation of EIF3M association with the tumor microenvironment

The TIMER2.0 database was employed to investigate the correlation between *EIF3M* expression and tumor-infiltrating immune cell abundance across pan-cancer cohorts. Processed data derived from the online analysis platform were extracted and subsequently visualized as a heatmap using GraphPad Prism (version 9.5). Relevant data from TCGA database were also extracted for StromalScore analysis, enabling further investigation into tumor microenvironment dysregulation caused by aberrant *EIF3M* gene expression.

### 2.6 Cell culture and transfection

The HCCLM3, MHCC97H and Hep3B were procured from Saiweier Biotechnology Co., Ltd. with authentication. All cell lines were maintained in DMEM supplemented with 10% fetal bovine serum (FBS, Gibco, 10091148) and 1% penicillin-streptomycin (Solaibio, P1400) in an incubator at 37 °C with 5% CO2. The coding sequences (CDS) of *EIF3M*,were inserted into the NheI/BamHI sites of the pcDNA3.1 plasmid. The siRNA duplex targeting *EIF3M*, sourced from GenePharma (A10001), was introduced into cells following strict adherence to the manufacturer’s transfection protocol to ensure precise delivery of the siRNA into the cellular interior. The siRNA sequences for the *EIF3M* negative control group and experimental group are detailed in Supplementary Table S1.

### 2.7 Quantitative real‐time polymerase chain reaction (qRT‐PCR)

Total RNA was extracted from cultured tumor cells using a total RNA extraction kit (Omega, R6834), and the concentration of the purified RNA was quantified using UV spectrophotometry. Following the manufacturer’s protocol of the reverse transcription kit, the extracted RNA was reverse-transcribed into complementary DNA (cDNA), which was subsequently amplified according to the instructions of the Plus All-in-one 1st Strand cDNA Synthesis SuperMix (gDNA Purge) (Novoprotein, E047-01B). The mRNA amplification reaction system is detailed in the Supplementary Table S2. The primer sequences for detecting the target gene *EIF3M* and the reference gene GAPDH in RT-qPCR experiments are listed in Supplementary Table S3.

### 2.8 Cell counting Kit-8

The Cell Counting Kit-8 (Servicebio, G4103-1 ML) was applied to determine tumor cell viability. Tumor cells were plated in 96-well plates and cultured under the aforementioned conditions. In compliance with the CCK-8 assay specifications, 10 μL of CCK-8 solution mixed with 90 μL culture medium was administered to each well at designated time intervals (0 h, 24 h, 48 h, 72 h, and 96 h). Following a 2-h incubation at 37 °C, absorbance values were recorded at 450 nm using a microplate reader for quantitative viability assessment.

### 2.9 Colony formation assay

Tumor cells were seeded at an ultra-low density in 6-well culture plates and maintained in a humidified 37 °C incubator with 5% CO_2_ atmosphere using complete growth medium. Cultures were continuously propagated for 7–14 days until microscopic visualization of colony formation. To sustain optimal cellular viability, medium replacement was performed every 3 days. At experimental termination, cells were gently rinsed with phosphate-buffered saline (PBS, Servicebio, G4202-500 ML) to eliminate non-viable cells, followed by fixation with 4% paraformaldehyde (Servicebio,G1101-500 ML) for 30 min. Subsequent staining was conducted using 0.1% crystal violet (Servicebio, G1014-50 ML) solution for 30 min. Post-staining, residual background dye was removed by slow-flow rinsing with deionized water. After air-drying, plates were imaged, and colony quantification was performed using ImageJ (version 1.54f) software through automated particle analysis.

### 2.10 Wound healing assay

Cells in logarithmic growth phase were seeded into culture dishes and incubated until reaching 80%–90% monolayer confluency. A standardized linear wound was mechanically introduced using a 200 μL sterile pipette tip held perpendicular to the dish surface to ensure uniform scratch width. Detached cells and cellular debris were removed by gentle washing with PBS. Initial images of the scratch wound were captured at 0 h using a microscope to establish baseline data. The culture dishes were subsequently maintained in a humidified 37 °C incubator with 5% CO_2_ atmosphere, and sequential images of the same microscopic fields were acquired at predetermined time points (24 h and 48 h). Post-experiment, image analysis was performed using ImageJ software to quantify the temporal changes in wound area.

### 2.11 Statistical analysis

In this study, selected foundational statistical computations were autonomously executed through online database systems. A variety of online platforms and tools employ distinct statistical methods to assess the significance of gene or protein expression, methylation levels, survival correlations, and molecular interactions. TIMER2.0 utilizes the edgeR algorithm by default to examine the significance of expression differences; UALCAN, based on the CPTAC database, applies Student’s t-test to compare protein-level and promoter methylation-level expression between cancerous and normal tissues; gene activity differential scores are typically evaluated using the Wilcoxon rank-sum test; GEPIA2 employs the Kaplan-Meier method, log-rank test, and Cox proportional hazards model for pan-cancer survival analysis; the Smart platform uses a one-sample t-test to analyze methylation level differences at specific CpG sites; and the ENCORI platform applies Spearman correlation analysis to assess the relationship between miRNA and *EIF3M*. For intergroup comparative analyses of cell phenotypic experimental data, a two-way analysis of variance (Two-way ANOVA) was implemented on the GraphPad Prism software. Results attaining this critical value were designated as demonstrating statistically significant differences. In this study, a threshold of p ≤ 0.05 was applied for all statistical comparisons to determine significant differences. The results of statistical analyses are annotated in the figures using the following symbols: ns (p > 0.05, not significant), * (p ≤ 0.05), ** (p ≤ 0.01), *** (p ≤ 0.001), and **** (p ≤ 0.0001).

## 3 Result

### 3.1 Pan-tissue expression and subcellular distribution

On the HPA integrated analysis platform, the RNA-seq data from HPA and GTEx databases were standardized and integrated to analyze the expression levels of EIF3M. Joint analysis revealed that *EIF3M* exhibits low tissue specificity, with no significant abnormal expression levels detected in any specific tissue among the 50 normal tissues (Supplementary Figure S1A). Furthermore, our analysis of EIF3M’s subcellular localization profile revealed its predominant localization within the cytosol (Supplementary Figure S1B). Immunofluorescence analysis of A-431, U2OS, and U-251MG tumor cells further corroborated this observation (Supplementary Figure S1C).

### 3.2 Comprehensive characterization of EIF3M expression across across human malignancies

**FIGURE 1:**
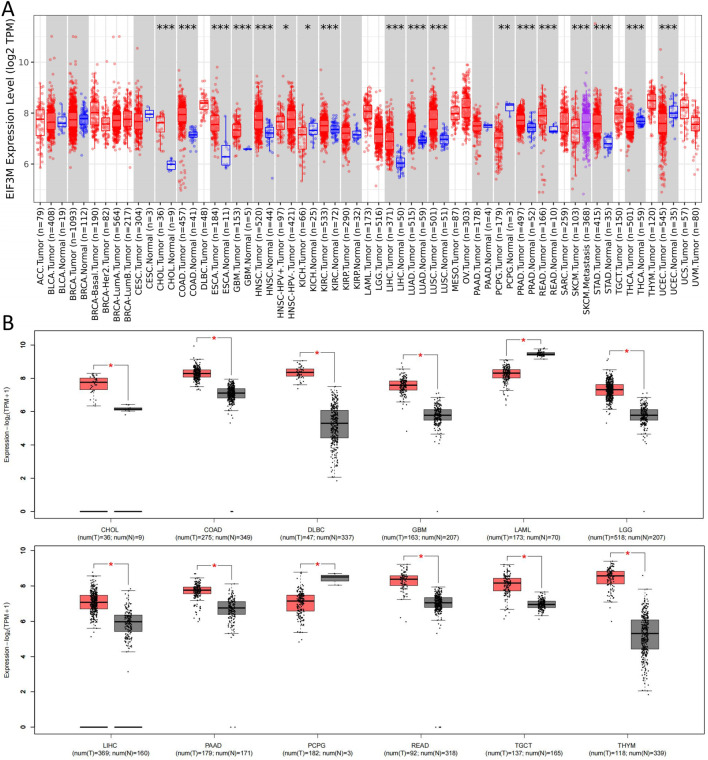
Expression levels of EIF3M across various tumor tissues. **(A)** On the TIMER2 platform, based on the TCGA database, the mRNA expression levels of EIF3M in various tumor tissues and specific tumor subtypes compared to normal tissues. **(B)** Corresponding analysis based on the TCGA database and GTEx datasets to analyze the expression differences of EIF3M mRNA levels between various tumor tissues and normal tissues.

**FIGURE 2:**
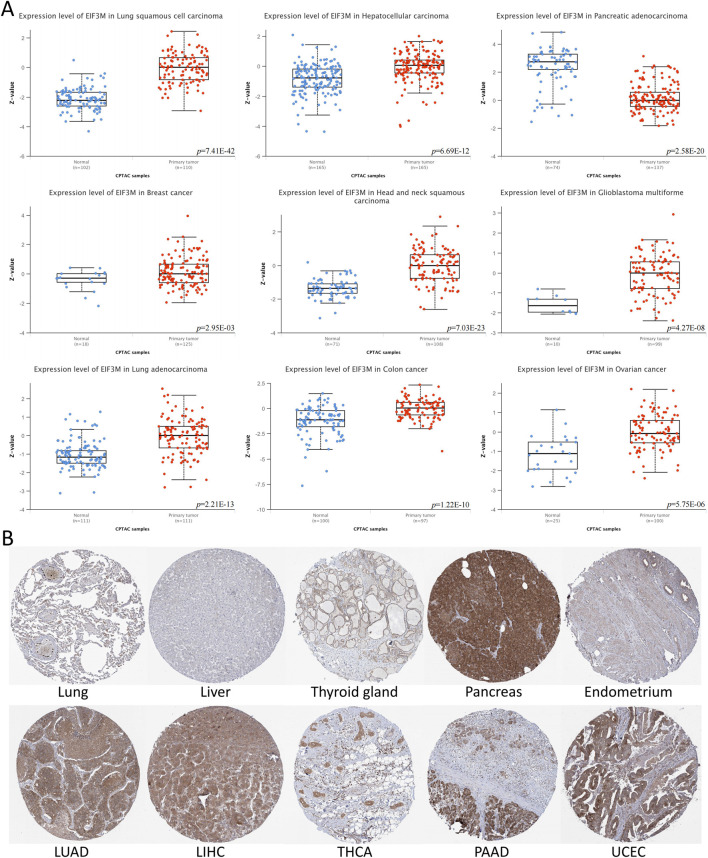
The protein expression levels of EIF3M across various tumor tissues. **(A)** Based on CPTAC data, analyze the protein expression differences between multiple tumor tissues and normal tissues. **(B)** Immunohistochemical images of EIF3M protein expression in multiple tumor and normal tissues.

Initially, we conducted a preliminary analysis of the expression levels of *EIF3M* in pan-cancer using the TIMER2.0 database based on TCGA data. The results demonstrated that *EIF3M* exhibited significantly elevated expression levels in the majority of human malignancies compared to their corresponding normal tissues (Figure 1A). To enhance the readability of the article, we have compiled the abbreviations and full names of all cancer types mentioned in this study, in accordance with the nomenclature standards of TCGA, as presented in Table 1. In CHOL, COAD, ESCA, GBM, HNSC, KIRC, LIHC, LUAD, LUSC, PRAD, READ, and STAD, the mRNA expression level of *EIF3M* exhibits a statistically significant upregulation across all these cancer types. We also observed that *EIF3M* expression was significantly downregulated in tumor tissues of KICH, PCPG, THCA, and UCEC. To address the limitation of limited availability of matched normal tissue expression data for certain cancer types within TCGA database, we propose an integrated analysis strategy combining data from TCGA and the Genotype-Tissue Expression (GTEx) project. This approach aims to more comprehensively evaluate the differential expression patterns of *EIF3M* across diverse tumor entities. The findings from this part of the study indicate that after further increasing the number of samples included in the research, *EIF3M* exhibited significantly higher expression levels in tumor tissues compared to normal tissues across the following cancer types: CHOL, COAD, DLBC, GBM, LGG, LIHC, PAAD, READ, TGCT, and THYM. However, in LAML and PCPG, *EIF3M* expression displayed the opposite trend, showing significantly lower levels in tumor tissues compared to normal tissues (Figure 1B). To further validate the expression characteristics of *EIF3M* at the post-transcriptional regulatory level, we systematically evaluated the protein-level expression patterns of *EIF3M* across multiple cancer types on the GEPIA2 platform using proteomics data from the CPTAC database. Through analysis, it has been observed that EIF3M exhibits significant expression abnormalities across multiple malignant tumor types. Specifically, in BRCA, COAD, GBM, HNSC, LIHC, LUAD, LUSC, and OV, protein expression levels of EIF3M demonstrate statistically significant upregulation compared to corresponding normal tissues. Conversely, in PAAD, a pattern of downregulated expression is observed. Through integrated analysis of immunohistochemistry data from the HPA database, we further validated the abnormal expression pattern of EIF3M (Figure 2A). Quantitative evaluation revealed statistically significant upregulation of EIF3M protein levels in LUAD, LIHC, THCA, PAAD, and UCEC compared with their corresponding normal tissues (Figure 2B). The immunohistochemistry sample information sourced from the HPA database is presented in Supplementary Table S4. Finally, to comprehensively characterize the transcriptional and regulatory networks of *EIF3M* in pan-cancer, we systematically integrated multi-dimensional omics data from the TCGA database for gene activity scoring analysis of *EIF3M*. Pan-cancer analysis revealed that *EIF3M* exhibited significantly elevated gene activity scores across 15 tumor types, including BLCA, BRCA, CHOL, COAD, ESCA, GBM, HNSC, KICH, KIRC, KIRP, LIHC, LUAD, PRAD, READ, and UCEC. Notably, in PCPG, the gene activity score was significantly lower compared to matched normal tissues, presenting a striking contrast (Supplementary Figure S2). The grouping and sample information utilized for each cancer type in the gene activity score analysis are presented in Supplementary Table S5.

**TABLE 1:** The cancer types mentioned in the article.

Abbreviation	Full name	Abbreviation	Full name
ACC	Adrenocortical carcinoma	LUAD	Lung adenocarcinoma
BLCA	Bladder Urothelial Carcinoma	LUSC	Lung squamous cell carcinoma
BRCA	Breast invasive carcinoma	OV	Ovarian serous cystadenocarcinoma
CHOL	Cholangiocarcinoma	PAAD	Pancreatic adenocarcinoma
COAD	Colon adenocarcinoma	PCPG	Pheochromocytoma and Paraganglioma
DLBC	Lymphoid Neoplasm Diffuse Large B-cell Lymphoma	PRAD	Prostate adenocarcinoma
ESCA	Esophageal carcinoma	READ	Rectum adenocarcinoma
GBM	Glioblastoma multiforme	SARC	Sarcoma
HNSC	Head and Neck squamous cell carcinoma	SKCM	Skin Cutaneous Melanoma
KICH	Kidney Chromophobe	STAD	Stomach adenocarcinoma
KIRC	Kidney renal clear cell carcinoma	TGCT	Testicular Germ Cell Tumors
KIRP	Kidney renal papillary cell carcinoma	THCA	Thyroid carcinoma
LAML	Acute Myeloid Leukemia	THYM	Thymoma
LGG	Brain Lower Grade Glioma	UCEC	Uterine Corpus Endometrial Carcinoma
LIHC	Liver hepatocellular carcinoma	UVM	Uveal Melanoma

### 3.3 Prognostic value of EIF3M in various tumors

**FIGURE 3:**
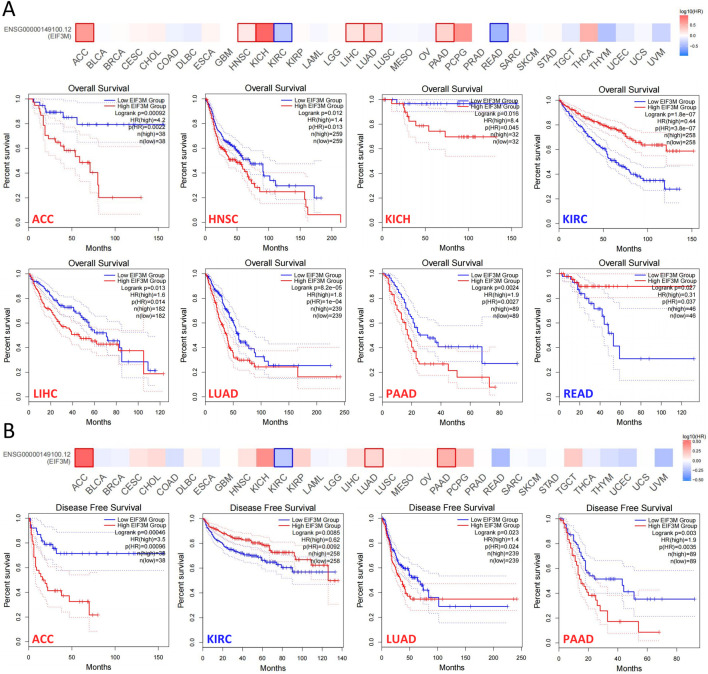
The correlation between EIF3M expression levels and patient survival rates. The analysis of EIF3M expression in relation to **(A)** overall survival and **(B)** disease-free survival in patients was conducted using the TCGA database via the GEPIA2 platform.

Tumor staging serves as one of the most pivotal indicators for predicting prognosis in cancer patients, with later-stage disease typically correlating with shorter survival duration and elevated recurrence risk (Sung et al., 2021). Therefore, we investigated the correlation between *EIF3M* expression levels and patients’ clinical staging. In KIRP, LIHC, and LUAD, higher tumor stages demonstrated a significant positive correlation with elevated *EIF3M* expression levels, while in SKCM, an opposing negative correlation trend was observed (Supplementary Figure S3A). To systematically evaluate the biological significance of *EIF3M* in prognosis across multiple cancer types, we performed survival curve analysis using the GEPIA2 database to investigate the clinical relevance between its expression levels and patient survival outcomes. In this part of the study, we stratified *EIF3M* expression levels into high and low groups using the median value as the cutoff. Our analysis revealed that elevated *EIF3M* expression was significantly associated with shorter overall survival (OS) in patients with ACC, HNSC, KICH, LIHC, LUAD, and PAAD. Conversely, an opposite prognostic trend was observed in KIRC and READ, where high *EIF3M* expression correlated with improved survival outcomes (Figure 3A). Pan-cancer analysis of disease-free survival (DFS) revealed that elevated *EIF3M* expression was significantly associated with shortened DFS in patients with ACC, LUAD, and PAAD. Conversely, an inverse correlation was observed in KIRC, where high *EIF3M* expression correlated with prolonged DFS (Figure 3B). To conduct a comprehensive analysis of the association between *EIF3M* expression levels and survival outcomes, we utilized data from TCGA to supplement our investigation into correlations between *EIF3M* expression and disease-specific survival (DSS) as well as progression-free survival (PFS) across pan-cancer cohorts. The analysis revealed that elevated *EIF3M* expression correlates with significantly shorter DSS in ACC, KICH, LIHC, LUAD, and PCPG. Conversely, in THYM and UVM, elevated *EIF3M* expression was associated with prolonged DSS (Supplementary Figure S3B). We also observed a significant correlation between *EIF3M* expression levels and PFS in patients with various malignant tumors. Survival analysis revealed that patients with high *EIF3M* expression exhibited significantly shorter PFS in ACC, KICH, KIRP, LIHC, LUAD, and PAAD. Conversely, in UVM and SKCM, high *EIF3M* expression demonstrated a trend toward improved prognostic outcomes (Supplementary Figure S3C). Detailed data on hazard ratios (HR) with 95% confidence intervals and p‑values for DSS and PFS analyses are provided in Supplementary Table S6.

### 3.4 The gene mutation and epigenetic modification of EIF3M

**FIGURE 4:**
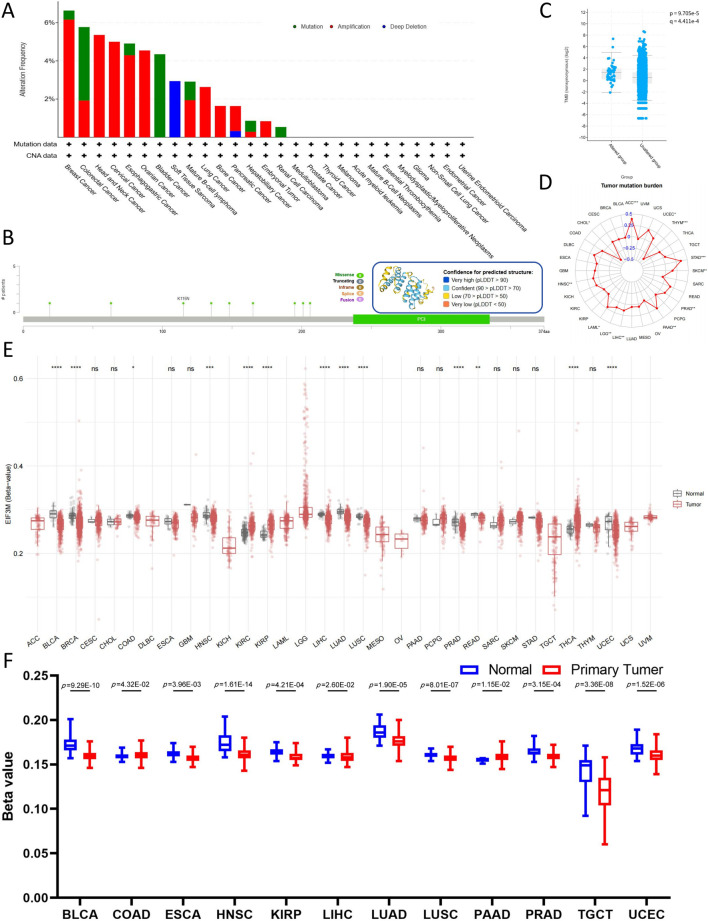
Mutations and epigenetic alterations of EIF3M in pan-cancer cells. **(A)** Mutation frequency and types of EIF3M. **(B)** Nine missense mutation sites in the EIF3M gene. **(C)** Tumor Mutational Burden score comparison between EIF3M-mutated and EIF3M-unmutated tumor groups. **(D)** Expression level differences of EIF3M in relation to Tumor Mutational Burden score across pan-cancer studies. **(E)** Differential expression of EIF3M in tumor tissues compared to normal tissues and its association with methylation alterations. **(F)** Analyze promoter methylation level differences in tumor tissues compared to normal tissues based on data from the UALCAN database.

Firstly, utilizing the cBioPortal analysis platform, select pan-cancer whole genome data from TCGA and the International Cancer Genome Consortium (ICGC) to construct the genetic alteration profile of *EIF3M*. Analysis of cancer types with sample sizes exceeding 10 cases revealed that BRCA, COAD, and HNSC exhibited the highest mutation frequencies. The *EIF3M* exhibits heterogeneous mutation frequencies across different cancer types: its highest mutation rate is observed in BRAC (6.64%), predominantly driven by Amplification (6.16%) with a minor contribution from Mutations (0.47%). In COAD, the overall mutation frequency is 5.77%, where Mutations account for a higher proportion (3.85%) compared to Amplification (1.92%). In HNSC, all mutational events are exclusively attributed to Amplification (5.36%) (Figure 4A). We also identified 9 missense mutation sites on *EIF3M* (Figure 4B). The mutation site sample information based on the cBioPortal platform is summarized in Supplementary Table S7. In-depth analysis revealed that patients harboring *EIF3M* alterations exhibited significantly higher tumor mutational burden (TMB) levels compared to those without *EIF3M* alterations (Figure 4C). To systematically evaluate the association between *EIF3M* expression and TMB, we stratified TCGA cohorts into high- and low-expression groups based on median *EIF3M* expression levels. The analysis revealed significant positive correlations in ACC, CHOL, HNSC, LAML, LGG, LIHC, PAAD, PRAD, SKCM, STAD, THYM, and UCEC (Figure 4D). In extended analyses, we systematically evaluated the DNA methylation profiles of *EIF3M* across pan-cancer cohorts, unveiling its epigenetic regulatory landscape. Comprehensive analysis revealed that CpG-dense regions displayed hypomethylation in BLCA, BRCA, COAD, HNSC, LIHC, LUAD, LUSC, PRAD, and READ compared to matched adjacent normal tissues. However, in KIRC, KIRP, THCA, and UCEC, CpG-dense regions exhibited significantly elevated methylation levels (Figure 4E). Promoter methylation, serving as one of the central mechanisms in epigenetic regulation, plays a pivotal role in transcriptional control. Pan-cancer analysis conducted through the ULCAN platform revealed hypomethylation in promoter regions of BLCA, ESCA, HNSC, KIRP, LIHC, LUAD, LUSC, PRAD, TGCT, and UCEC compared to matched adjacent normal tissues, while COAD and PAAD demonstrated significant hypermethylation (Figure 4F). The grouping and sample information for CpG-dense regions and promoter methylation across various cancer types are presented in Supplementary Tables S8, 9.

### 3.5 Functional and pathway enrichment analysis of genes related to EIF3M

Based on the Pathway Commons biological pathway integration analysis platform, the characteristics of the *EIF3M* interaction network were systematically analyzed through standardized annotation of multi-source molecular interaction data. A curated set of candidate genes exhibiting significant interactions with *EIF3M* was identified, including EIF5, METTL3, PRPF8, DHRS2, ESR2, STOML2, EEF2, ESR1, SEPTIN7, PSMD12, PRNP, SEMA7A, ODF2, RECQL4, G3BP1, SMN1, SMN2, UBB, ANGEL1, G3BP2, NP2C2, ANXA1, SAFB, and PSPC1 (Supplementary Figure S4A). Based on the GEPIA2 analysis platform, a systematic evaluation of the expression profiles of the aforementioned gene set in pan-cancer and normal tissues was conducted. The results demonstrated that EEF2, G3BP1, G3BP2, PRPF8, RECQL4, STOML2, and UBB exhibited significantly higher expression levels in the majority of tumor tissues, whereas METTL3 and NR2C2 showed a widespread downregulation trend (Supplementary Figure S4B). To further elucidate the underlying biological functions and regulatory pathways in which the aforementioned gene set may be involved, this gene set was submitted to the Enrichr online analysis platform for enrichment analysis. In pathway enrichment analyses analysis, *EIF3M*-associated gene sets are closely related to biological processes such as gene expression, nuclear receptors and nuclear receptors transcriptional pathways in BioPlanet. Reactome pathway analysis revealed that these genes were significantly enriched in the Nuclear Receptor Transcription Pathway, Regulation of RUNX2 Expression and Activity, and MAPK6 MAPK4 Signaling (Supplementary Figure S4C). Ontolo

gical analysis revealed that the *EIF3M* gene set demonstrated significant enrichment in specific biological processes and molecular functions. Within the GO Biological Process category, this gene set exhibited pronounced enrichment in estrogen receptor signaling pathway, regulation of stress granule assembly and membraneless organelle assembly. Concurrently, in the GO Molecular Function domain, it showed marked enrichment profiles for estrogen response element binding, nuclear steroid receptor activity and mRNA binding capabilities (Supplementary Figure S4D). STRING is an essential bioinformatics platform for studying protein-protein interactions (PPI). On this platform, 10 proteins with high interaction with EIF3M have been identified: EIF3A, EIF3B, EIF3C, EIF3D, EIF3E, EIF3F, EIF3G, EIF3H, EIF3I and EIF3K (Supplementary Figure S4E). Gene Ontology (GO) analysis demonstrated significant enrichment in the molecular function category for terms including translational initiation activity, RNA binding, translation initiation factor binding, and metal-dependent deubiquitinase activity. Within biological processes, the most prominently enriched term was formation of cytoplasmic translation initiation complex (Supplementary Figure S4F).

### 3.6 Bioinformatic prediction and functional enrichment analysis of EIF3M-Regulating miRNAs

**FIGURE 5:**
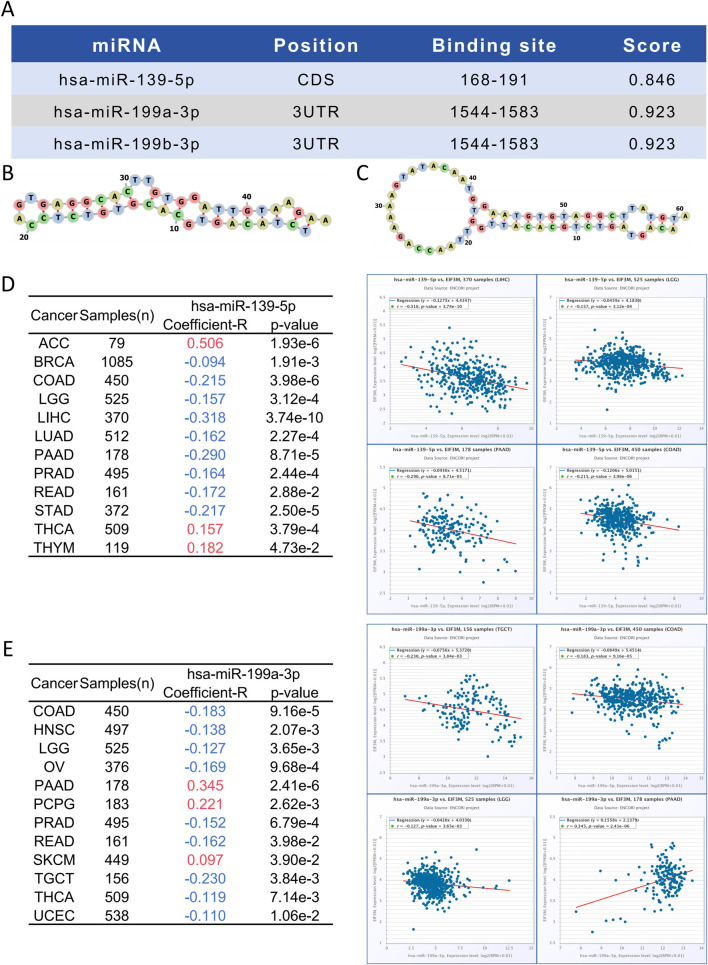
Identification of miRNAs binding sites and pan-cancer analysis of their correlation with EIF3M expression. **(A–C)** Prediction of miRNA binding sites targeting EIF3M. Correlation between **(D)** hsa-miR-139-5p and **(E)** hsa-miR-199a-3p with EIF3M expression across various cancers.

miRNAs serve as key regulators in post-transcriptional regulation, and fluctuations in their expression levels can profoundly influence target gene expression. Therefore, investigating miRNAs targeting *EIF3M* holds substantial research significance. A tri-platform screening strategy integrating miRDB, miRWalk, and TargetScan predictions was employed to identify *EIF3M*-targeting miRNAs. Venn analysis demonstrated 41 consensus miRNAs shared across all databases, representing a high-confidence candidate set with multi-algorithmic validation (Supplementary Figure S5A). Using pan-cancer analysis heatmaps generated by the dbDEMC platform, we identified hsa-miR-139-5p, hsa-miR-199a-3p and hsa-miR-199b-3p as candidate miRNAs through expression profiling screening (Supplementary Figure S5B). Bioinformatics analysis revealed that hsa-miR-139-5p targets the CDS region of *EIF3M*, while hsa-miR-199a-3p and hsa-miR-199b-3p specifically interact with its 3′UTR (Figure 5A). Detailed complementary sequences and predicted secondary structures for these miRNA-*EIF3M* interactions are shown in Figure 5B-C. Next, these three filtered core miRNAs were imported into the ENCORI platform to further investigate whether their expression patterns are correlated with *EIF3M* across pan-cancer contexts. The results revealed that, among the 12 cancer types where hsa-miR-139-5p exhibited significant correlation with *EIF3M* expression, 9 cancers (BRAC, COAD, LGG, LIHC, LUAD, PAAD, PRAD, READ, STAD) displayed a negative correlation in their expression patterns (Figure 5D). For hsa-miR-199a-3p and hsa-miR-199b-3p, among the 12 cancer types showing significant correlation with *EIF3M*, 9 cancers (COAD, HNSC, LGG, OV, PRAD, READ, TGCT, THCA, UCEC) demonstrated a negative correlation in their expression (Figure 5E; Supplementary Figure S6A). To elucidate the molecular mechanisms and underlying biological processes of hsa-miR-139-5p, hsa-miR-199a-3p, and hsa-miR-199b-3p, we systematically conducted functional enrichment analysis utilizing the ENCORI database. KEGG pathway and Disease Ontology analyses revealed that, out of the 10 significantly associated pathways for hsa-miR-139-5p, 6 were directly linked to cancer. Notably, this miRNA also exhibited regulatory associations with the p53 signaling pathway and focal adhesion pathway—both critically implicated in tumorigenesis. Further GO functional module analysis demonstrated that at the Biological Process, this molecule primarily enriched in biological metabolism-related processes, while at the Molecular Function, it showed significant involvement in RNA-binding (Table 2). KEGG pathway and disease ontology analyses revealed that hsa-miR-199a-3p and hsa-miR-199b-3p are significantly enriched in cancer-related signaling pathways. Additionally, GO functional analysis indicated that, at the levels of biological processes and molecular functions, these two miRNAs are primarily involved in metabolic biosynthesis processes, nucleic acid binding, and transcriptional regulation functions (Table 3; Supplementary Table S10).

**TABLE 2:** Top five miRNA-target interactions in enrichmentanalysis of hsa-miR-139-5p.

pathwayName	log10(p-val)	log10(FDR)
KEGG		
KEGG_Pathways_In_Cancer	−26.28905	−24.07157
KEGG_P53_Signaling_Pathway	−13.50613	−11.58968
KEGG_Neurotrophin_Signaling_Pathway	−13.28473	−11.54437
KEGG_Small_Cell_Lung_Cancer	−12.86315	−11.42381
KEGG_Focal_Adhesion	−13.03558	−11.42015
Disease ontology		
Of_Gene-disease_Association	−46.5856	−43.78903
In_Breast_Cancer	−35.1121	−32.61655
Of_Cancer	−31.69575	−29.3763
In_Tumors	−29.8869	−27.69239
In_Prostate_Cancer	−19.47485	−17.37725
Biological processe		
GOBP_Negative_Regulation_Of_Biosynthetic_Process	−88.24621	−84.78494
GOBP_Negative_Regulation_Of_Nucleobase_Containing_Compound_Metabolic_Process	−88.41281	−84.65051
GOBP_Positive_Regulation_Of_Biosynthetic_Process	−87.6268	−84.34162
GOBP_Cellular_Macromolecule_Localization	−85.7862	−82.72287
GOBP_Positive_Regulation_Of_Nucleobase_Containing_Compound_Metabolic_Process	−85.80708	−82.64683
Molecular function		
GOMF_Enzyme_Binding	−87.98419	−84.93381
GOMF_Transcription_Regulator_Activity	−74.3217	−71.57235
GOMF_Rna_Binding	−70.21378	−67.64052
GOMF_Sequence_Specific_Dna_Binding	−63.87299	−61.42467
GOMF_Ribonucleotide_Binding	−58.92984	−56.57843

**TABLE 3:** Top five miRNA-target interactions in enrichmentanalysis of hsa-miR-199a-3p.

pathwayName	log10(p-val)	log10(FDR)
KEGG		
KEGG_Mapk_Signaling_Pathway	−18.92645	−16.71426
KEGG_Pathways_In_Cancer	−17.77964	−15.86848
KEGG_Focal_Adhesion	−17.2644	−15.52934
KEGG_Regulation_Of_Actin_Cytoskeleton	−16.42243	−14.81231
KEGG_Neurotrophin_Signaling_Pathway	−15.26997	−13.75675
Disease ontology		
Of_Gene-disease_Association	−41.53348	−38.78607
In_Tumors	−27.73465	−25.28827
In_Breast_Cancer	−26.93135	−24.66106
In_Prostate_Cancer	−19.16659	−17.02123
Of_Cancer	−17.2255	−15.17706
Biological processe		
GOBP_Positive_Regulation_Of_Nucleobase_Containing_Compound_Metabolic_Process	−76.63465	−72.89516
GOBP_Positive_Regulation_Of_Biosynthetic_Process	−75.26942	−71.83096
GOBP_Cellular_Macromolecule_Localization	−73.59791	−70.33554
GOBP_Regulation_Of_Intracellular_Signal_Transduction	−68.97741	−65.83997
GOBP_Regulation_Of_Protein_Modification_Process	−66.87656	−63.83603
Molecular function		
GOMF_Enzyme_Binding	−68.4878	−65.45357
GOMF_Transcription_Regulator_Activity	−55.59786	−52.86466
GOMF_Identical_Protein_Binding	−52.29043	−49.73332
GOMF_Rna_Binding	−49.7899	−47.35773
GOMF_Sequence_Specific_Dna_Binding	−47.83628	−45.50103

### 3.7 Analysis of immune infiltration characteristics of EIF3M in the tumor microenvironment

**FIGURE 6:**
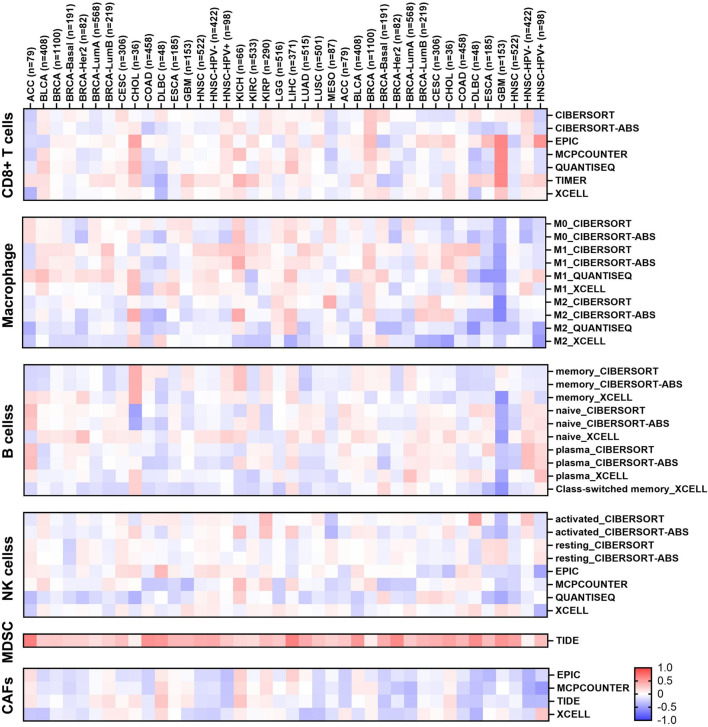
Heatmap of the correlation between EIF3M expression and infiltration of various immune cells.

The study of TIME represents a central paradigm in the fields of cancer biology and immunotherapy. To investigate whether aberrant expression of *EIF3M* induces alterations in immune cell levels, we conducted further exploration using the TIMER 2.0 data analysis platform. As shown in Figure 6, cancer types showing significant positive correlations with B cells include THCA, Sarcoma (SARC), PRAD, KIRC, and LIHC, primarily involving plasma cells and memory B cell subsets. Notable negative correlations are observed in KIRC, LGG, THYM, and LUAD. For cancer-associated fibroblasts (CAFs), LIHC exhibits the only significant positive correlation, while BLCA, HNSC, LUSC, PRAD, SARC, THCA, and UVM show significant negative correlations. CD8^+^ T cells demonstrate strong positive correlations with THYM, SKCM, UVM, PCPG, and LIHC, but negative correlations with PRAD, TGCT, LUSC, and KIRC. M1 macrophages display significant positive associations with BRCA, HNSC, and LUAD, but a strong negative correlation with THCA. Conversely, M2 macrophages show positive correlation with LIHC and negative correlations with KIRC, LUAD, and THCA. All significant MDSC correlations are positive, including LIHC, COAD, HNSC, and LUAD. NK cells exhibit significant negative correlations with LUAD, LGG, THCA, COAD, GBM, and PRAD, but positive correlations with KIRP, TGCT, and LIHC. We evaluated the immune scores of *EIF3M* in pan-cancer using the TCGA database. StromalScore demonstrated correlations with *EIF3M* expression across multiple cancer types, including ACC, BRCA, COAD, GBM, HNSC, LAML, LGG, LIHC, LUAD, OV, PAAD, PRAD, READ, SARC, SKCM, STAD, THCA, UCEC, and UVM. Conversely, for ImmuneScore, statistically significant correlations with *EIF3M* expression were observed only in ACC, CHOL, COAD, DLBC, LAML, LGG, OV, READ, SKCM, THCA, and UVM as shown in Supplementary Table S11.

### 3.8 EIF3M promotes HCC progression by enhancing proliferation and migration

**FIGURE 7:**
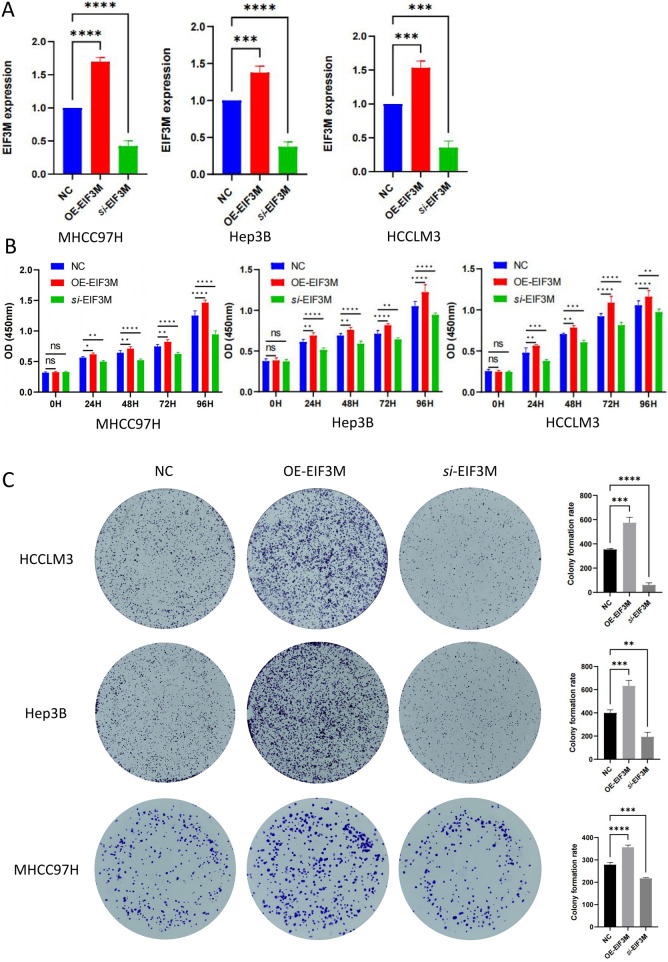
The impact of EIF3M expression on the proliferative capacity of HCC cell lines. **(A)** RT-qPCR validation of EIF3M overexpression and knockdown models in three HCC cell lines. Results of **(B)** CCK-8 assay and **(C)** colony formation assay for three cell lines with differential EIF3M expression levels *in vitro*.

**FIGURE 8:**
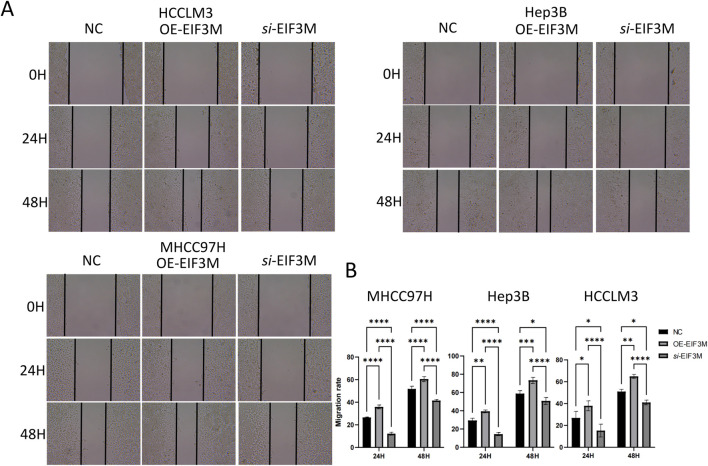
Results of wound healing assay. Results of **(A)** wound healing assay and corresponding **(B)** statistical analysis for the three cell lines with differential EIF3M expression levels.

Through integrated analysis of multi-omics data, we identified a significant correlation between *EIF3M* expression dysregulation and tumor progression, suggesting its functional involvement in this process. To elucidate the functional role of EIF3M in tumor progression, we assessed the regulatory effects of its dysregulated expression on the core biological behaviors of tumor cells. To address this objective, this study employed well-characterized HCC cell lines as experimental models to investigate and elucidate the critical knowledge gap regarding the functional role of *EIF3M* expression patterns in the biological behavior of HCC. This study initially established *EIF3M* knockdown and overexpression cell models in three typical hepatocellular carcinoma cell lines (MHCC97H, Hep3B, and HCCLM3), and systematically verified gene expression levels at the mRNA level using qRT-PCR, thereby laying a reliable experimental foundation for subsequent functional studies (Figure 7A). To investigate the impact of *EIF3M* expression levels on the proliferative activity of HCC cells, functional validation was conducted in this study using the CCK-8 cell proliferation assay and colony formation assay. The experimental results demonstrated that *EIF3M* overexpression significantly enhanced the proliferative capacity of hepatocellular carcinoma cells, while *EIF3M* knockdown resulted in a marked reduction in tumor cell proliferation (Figure 7B). Furthermore, *EIF3M* expression level exhibited a positive correlation with colony formation efficiency, as evidenced by the significantly increased number of cell colonies formed in the overexpression group. Conversely, attenuation of *EIF3M* expression led to pronounced inhibition of colony-forming ability (Figure 7C). Simultaneously, the impact of *EIF3M* expression on cell migration capacity was evaluated, revealing a positive correlation between migration ability and *EIF3M* expression levels. Scratch wound healing assays demonstrated that *EIF3M* overexpression significantly promoted wound closure rate. Conversely, suppression of *EIF3M* expression resulted in marked inhibition of cellular migratory activity, accompanied by prolonged wound healing duration (Figure 8A,B). To investigate the potential mechanisms by which EIF3M influences HCC cells, we performed KEGG pathway analysis. Among the top ten pathways ranked by enrichment score (Supplementary Table S12), the KEGG_WNT_SIGNALING_PATHWAY was identified as highly relevant to the initiation, progression, and malignant behavior of HCC. To explore potential interacting factors, we intersected the EIF3M-correlated gene set (Supplementary Table S13) with genes enriched in the KEGG_WNT_SIGNALING_PATHWAY, which led to the identification of FZD2 as a key candidate regulator.

## 4 Discussion

EIF3M, as a core structural subunit of the eIF3 complex, exhibits widespread expression of its encoded gene product across various tissues. It plays a critical regulatory role in the initiation stage of eukaryotic protein synthesis by mediating the assembly process of the 43S pre-initiation complex (43S PIC) (Gomes-Duarte et al., 2018). Recent studies have progressively unveiled the critical regulatory role of the EIF3M in tumorigenesis and progression. Its dysregulated expression has been substantiated to correlate with malignant progression and adverse clinical outcomes in various solid tumors, including but not limited to LUAD, LUSC, PRAD, and BRCA (9,10,12). However, the regulatory mechanisms governing EIF3M’s dysregulated expression in cancer and its associated biological functions remain poorly understood, necessitating further in-depth investigation (Yin et al., 2018). To bridge this gap, our pan-cancer multi-omics analysis revealed that EIF3M is frequently overexpressed and genomically altered across cancer types, which correlates with unfavorable patient outcomes. We further elucidated its involvement in epigenetic modulation, miRNA-mediated regulatory networks, and remodeling of the TIME. These findings not only deepen the understanding of EIF3M’s oncogenic role but also highlight its potential as a prognostic biomarker and therapeutic target. To functionally validate these observations, we have successfully established both *EIF3M* overexpression and knockdown cellular models. Functional experiments using these models have confirmed that EIF3M significantly promotes tumor cell proliferation and migration.

In the present study, based on transcriptome data analysis from TCGA and GTEx databases, identified significant differential mRNA expression of the *EIF3M* across 12 tumor types: CHOL, COAD, DLBC, GBM, LGG, LIHC, PAAD, READ, TGCT, THYM, LAML, and PCPG. Furthermore, protein expression analysis revealed significant differential expression of this gene in 9 malignancies: BRCA, COAD, GBM, HNSC, LIHC, LUAD, LUSC, OV, and PAAD. Integrated analysis revealed concordant upregulation of EIF3M expression at both mRNA and protein levels in COAD, GBM, and LIHC. This dual aberrant overexpression pattern suggests its potential role as a critical driving factor in the pathogenesis of these three malignancies. Furthermore, the cross-omics expression profile demonstrates promising utility as a high-performance diagnostic biomarker for these cancers. This study also uncovered a unique molecular expression signature of *EIF3M* in PAAD: its mRNA levels exhibit an inverse relationship with protein abundance compared to normal tissues. This paradoxical regulatory pattern suggests *EIF3M* may undergo complex post-transcriptional control mechanisms, including miRNA-mediated translational repression and ubiquitin-proteasome system-dependent protein degradation. These regulatory pathways likely underpin its pro-tumorigenic role in cancer initiation and progression. Notably, this discovery provides a critical molecular framework for developing precision therapeutic strategies targeting *EIF3M* in oncology, holding substantial promise for clinical translation. To explore evidence supporting EIF3M as a novel tumor diagnostic marker and prognostic predictor, we conducted an exploration and discussion by systematically analyzing the correlation between *EIF3M* expression levels and tumor Stage. The results revealed that in KIRP, LIHC, and LUAD, *EIF3M* expression levels exhibited a positive correlation trend with tumor Stage, whereas *EIF3M* expression was significantly lower in advanced-stage SKCM patients compared to early-stage cases. These complex findings suggest that during tumor progression, genomic instability drives spatiotemporal fluctuations in EIF3M expression levels, which are dynamically regulated through multilayered networks to adapt to the tumor microenvironment. This adaptive process likely involves functional reprogramming of oncogenic mechanisms, enabling cancer cells to reshape their biological functions in response to evolutionary pressures (Jardim et al., 2025; Alonso et al., 2025). Through multidimensional survival analysis, we further evaluated the clinical association of *EIF3M* expression levels with OS, DFS, DSS, and PFS. After screening cancer types with ≥3 statistically significant survival indicators, we identified that high *EIF3M* expression in ACC and LUAD was significantly associated with poor patient prognosis. In KICH and LIHC, patients with elevated *EIF3M* levels exhibited worsening trends in OS, DSS, and PFS. Similarly, in PAAD, *EIF3M* overexpression demonstrated statistically significant inverse associations with OS, DFS, and PFS outcomes. The integrated analysis of expression-prognosis correlation patterns confirms that differential EIF3M expression serves as a pan-cancer diagnostic and prognostic biomarker (Zhou et al., 2025). To provide a comprehensive overview of the expression alterations of *EIF3M* across various cancer types and its association with patient prognosis, the corresponding findings are systematically summarized in Table 4. This discovery not only establishes a theoretical foundation for developing clinical prediction models but also provides a novel entry point for advancing the clinical translation of EIF3M-based therapeutic strategies, thereby bridging the evidence chain from mechanistic research to practical applications.

**TABLE 4:** Expression changes of EIF3M across cancers and their prognostic associations.

	Expression level	EIF3M expression		High expression & prognosis	
	Low expression	High expression		Protective factor	Risk factor
Cancer type	KICH	BRCA	LUAD	KIRC	ACC
	LAML	CHOL	LUSC	READ	HNSC
	PAAD	COAD	OV	THYM	KICH
	PCPG	ESCA	PRAD	SKCM	LIHC
	THCA	GBM	READ	UVM	LUAD
	UCEC	HNSC	STAD		PAAD
		KIRC			PCPG
		LIHC			KIRP

In tumor biology, the mutation frequency of oncogenes, DNA methylation levels, and miRNA-mediated regulatory networks collectively drive tumorigenesis and progression through distinct molecular layers (Yang et al., 2021). These interconnected mechanisms form the basis of tumor heterogeneity, and investigating these axes provides critical insights into the biological functions of genes in cancer development. Pan-cancer genomic analysis revealed that the *EIF3M* exhibits frequent genomic alterations across 15 major malignancies, with BRCA, COAD, and HNSC showing particularly high mutational rates. Amplification represents the predominant alteration type for this gene. Our pan-cancer TMB analysis identified a significant correlation between *EIF3M* expression levels and TMB status in 12 malignancies. Furthermore, tumors with *EIF3M* mutations exhibited significantly elevated TMB scores compared to wild-type counterparts. Integrating mutation frequency and TMB data enables the evaluation of genomic instability in patients, providing a scientific rationale for developing personalized therapeutic strategies (Zy et al., 2024; Sholl et al., 2020). CpG methylation analysis of the *EIF3M* revealed significant differences in methylation levels between 13 cancer types and normal tissues. Notably, promoter region assessment demonstrated aberrant methylation patterns in 12 of these malignancies. In most cancer types, *EIF3M* exhibits hypomethylation patterns relative to normal tissues, and this epigenetic alteration correlates with its consistent overexpression observed in tumors (Gomes-Duarte et al., 2018). This inverse relationship aligns with the well-established role of DNA methylation as a transcriptional repressor in cells (Zhou et al., 2025; Ji et al., 2022). MiRNAs play a pivotal role in gene expression regulation and may provide critical clues for elucidating novel regulatory mechanisms of oncogenes (Alexander et al., 2025). Our pan-cancer analysis identified three miRNAs that target *EIF3M* and show a significant negative correlation with its expression. In most cancer types, *EIF3M* expression is negatively correlated with these targeting miRNAs, which is consistent with its significant overexpression in tumor tissues compared to normal counterparts (Liu et al., 2021; Zhou et al., 2025). Integrative analysis of genetic mutations, methylation, and miRNAs transcends the limitations of the traditional “driver mutation” paradigm by enabling multi-layered integration of epigenetic-transcriptomic-genomic data. This approach uncovers the dynamic interplay among these three elements within regulatory networks, thereby offering a novel paradigm for cancer mechanism research, precision diagnostics, and targeted therapeutics.

Genes do not operate in isolation. Therefore, systematically investigating the coordinated regulatory mechanisms within complex oncogenic networks is crucial. It holds the potential to elucidate core molecular networks that govern oncogenic reprogramming by driver genes and immune evasion during tumorigenesis and progression. This understanding will provide a theoretical foundation for deciphering the evolutionary principles of tumor heterogeneity and progression dynamics (Singh et al., 2019). Using the Pathway Commons database, we identified a regulatory module of 24 genes with significant molecular interactions with EIF3M. We then systematically constructed a pan-cancer expression atlas for this gene set and their normal tissue counterparts. Transcriptomic profiling revealed that *EIF3M*-associated genes (e.g., EEF2, G3BP1, G3BP2, PRPF8, RECQL4, STOML2, and UBB) are widely dysregulated in tumors. GSEA revealed that this gene cohort is significantly enriched in functional modules related to transcriptional regulation and core signaling pathways. Next, we built a protein-protein interaction network using the STRING database and identified 10 core proteins that directly interact with EIF3M, all belonging to the EIF3 family. Functional annotation confirmed that this protein cluster is also highly enriched in GO terms associated with transcriptional regulation. The findings from this section demonstrate a multi-dimensional collaborative network map of EIF3M in tumorigenesis and progression, providing crucial evidence for systematically analyzing its mediated biological processes and precisely identifying key signaling axes. This analysis reveals the topological structure of the protein interaction network centered on EIF3M as a core node, which transcends the limitations of traditional single-factor studies. These results establish a foundation for deciphering the functional plasticity of EIF3M in the context of tumor heterogeneity and developing potential therapeutic targets.

The TIME, a complex ecosystem composed of tumor cells, surrounding immune cells, stromal components, and signaling molecules, serves not only as a pivotal key to understanding the essence of tumor biology but also as an indispensable pathway to transcend current therapeutic barriers and achieve precision medicine (Jin et al., 2023). By dissecting the intricate regulatory networks within TIME, we can furnish theoretical foundations for the development of novel immunocombination therapies, the overcoming of drug resistance, and the realization of individualized treatment strategies (Liu N. et al., 2025). Recent studies have progressively unveiled the central role of the EIF3 family in shaping the tumor immune microenvironment. Multiple EIF3 subunits—including B, D, and F—mediate immunosuppressive functions through multidimensional mechanisms such as exosomal sorting, metabolic reprogramming, and regulation of gene expression, collectively promoting immune microenvironment remodeling and resistance to immunotherapy (Zhang et al., 2025; Zhou et al., 2024; Lu et al., 2024). In studies focused on hepatocellular carcinoma and melanoma, elevated expression of specific EIF3 subunits (B and F) has been demonstrated to directly or indirectly suppress CD8^+^ T cell infiltration and impair the efficacy of PD-1 blockade therapy (Zhou et al., 2025; Wu et al., 2022). The present study also revealed that EIF3M, as a member of the EIF3 gene family, exhibits a significant correlation between its aberrant expression and dynamic alterations in immune cell infiltration density within the TIME. Under pathological conditions, dysregulation of this gene is accompanied by pronounced changes in the infiltration levels of multiple immune cell subsets, including CD8^+^ T cells, macrophages, B cells, NK cells, MDSC, and CAFs. CD8^+^ T lymphocytes, NK cells, and M1-polarized macrophages collectively function as central effector units within the immune system, mediating antitumor immunity through direct cytolytic elimination of malignant cells and paracrine secretion of immunoregulatory cytokines/chemokines, thereby constituting pivotal operational components of the immune surveillance network (Xing et al., 2025; Park et al., 2024; Palmer et al., 2025). Under conditions of EIF3M overexpression, functional suppression of immune cells may be associated with metabolic reprogramming. For instance, in HCC, depletion of polyunsaturated fatty acids (PUFAs) leads to diminished antitumor cytotoxicity of immune cells, thereby contributing to resistance to immunotherapy (An and Li, 2025). M2-type macrophages, MDSCs, and CAFs collectively orchestrate the establishment of an immunosuppressive microenvironment and remodeling of tumor stromal architecture through the secretion of immunosuppressive cytokines, chemokines, and extracellular matrix components (Jin et al., 2023; Zhu et al., 2023; Oya et al., 2020). In this study, it was observed that high expression of *EIF3M* is accompanied by significantly elevated levels of MDSCs across nearly all cancer types. This suggests that EIF3M may promote the formation of an immunosuppressive microenvironment potentially through the IL-6/CD73 axis, thereby modulating the activity of other immune cells (Liu M. et al., 2025). Furthermore, elucidating the molecular interaction mechanisms between EIF3M and immune cell subsets not only establishes the molecular biological foundation for developing dynamic immune score monitoring models and prognostic stratification systems, but also offers novel insights into the exploration of immunotherapy-based combination strategies and provides an innovative research perspective for addressing current clinical therapeutic challenges (Zhou et al., 2025).

In cancer functional genomics research, modulating the expression levels of oncogenes in tumor cell lines to investigate their regulatory effects on cellular biological behaviors represents a core research strategy widely employed in this field (Gong et al., 2025). To elucidate the cellular-level biological functions of EIF3M, this study established both knockdown and overexpression models of *EIF3M* in HCC cell lines and conducted systematic functional validation experiments. Through systematic evaluation utilizing CCK-8 cell proliferation assays and colony formation experiments, we demonstrated that downregulation of *EIF3M* gene expression significantly suppresses proliferative activity in LIHC malignant tumor cells, whereas overexpression of *EIF3M* markedly enhances proliferative capacity across HCC cell lines. Further analysis revealed that this regulatory effect also exerts significant biological impacts on migratory phenotypes: Wound healing assays indicated that suppression of *EIF3M* expression resulted in markedly reduced migratory capacity in tumor cells of the knockdown group compared to control counterparts, while the overexpression group exhibited a contrasting phenotype characterized by significantly enhanced migratory ability. These findings collectively suggest that EIF3M may contribute to malignant tumor progression by concurrently regulating critical cellular processes involving proliferation and migration. KEGG enrichment analysis revealed a significant positive association between EIF3M and the Wnt signaling pathway. Notably, FZD2—a gene correlated with EIF3M—was also enriched within this pathway. These observations suggest that EIF3M may activate the Wnt signaling cascade through the modulation of FZD2 expression, thereby promoting malignant phenotypes in HCC cells, including proliferation, invasion, and metastasis. Research on EIF3M has not only expanded our understanding of tumor biological behaviors but also provided critical insights for the development of innovative diagnostic tools and therapeutic strategies. Through multidisciplinary integration and deep functional characterization, continued investigation into its mechanistic roles holds significant potential to catalyze transformative progress in precision oncology.

Although the present study has conducted a systematic investigation into the potential value of EIF3M as a novel biomarker in pan-cancer analysis, the development of clinical prognostic evaluation systems, and the exploration of its molecular regulatory mechanisms, it is imperative to objectively acknowledge several inherent limitations of the current research. Specifically, this study primarily relied on retrospective analyses of public omics databases, and the correlations between relevant molecular signatures and clinical indicators still necessitate validation through prospective clinical cohort studies to confirm their clinical translational validity. It should be noted that although this study has preliminarily validated the functional roles of EIF3M in regulating malignant phenotypes in HCC through *in vitro* models, its cross-cancer applicability still requires confirmation through systematic functional genomic studies. Furthermore, the multidimensional biological effects mediated by EIF3M and its associated molecular regulatory networks still necessitate systematic dissection through multi-layered functional genomics experiments. The translational medical value of EIF3M is of critical importance; however, its practical application remains highly contingent upon further validation and in-depth exploration through prospective clinical cohort studies and *in vivo* experiments. Despite the aforementioned limitations, this study has offered significant insights into elucidating the mechanistic role of EIF3M in tumorigenesis and its clinical translational potential. Firstly, through multi-dimensional data validation, we have confirmed the potential association between EIF3M expression patterns and malignant tumor phenotypes, providing critical targets for subsequent functional verification. Secondly, we have preliminarily mapped the signaling pathway regulatory networks potentially involving EIF3M, establishing a research foundation for clarifying its molecular mechanisms. Finally, employing bioinformatics analysis combined with phenotype correlation studies based on HCC cell lines, we have provided theoretical underpinnings for designing targeted intervention strategies and developing novel prognostic biomarkers. These discoveries not only expand the functional understanding of the eukaryotic translation initiation factor family in tumor biology but also highlight the translational medical significance of EIF3M as a potential therapeutic target.

## 5 Conclusion

This study systematically analyzed the expression profile of EIF3M in pan-cancer tissues and revealed its significant overexpression in multiple malignancies such as LIHC, COAD, and LUAD. EIF3M expression was closely associated with poor patient prognosis, including reduced OS and DFS, as well as key biological processes such as genetic mutations, TMB, DNA methylation, miRNA regulatory networks, and remodeling of the tumor immune microenvironment. Furthermore, using HCC cell line models, this study demonstrated that differential *EIF3M* expression levels markedly influence tumor cell behaviors, including proliferation and migration. While the conclusions are primarily supported by multi-omics analyses from public databases, the biological functions and clinical significance of EIF3M warrant further validation through in-depth mechanistic investigations and clinical studies.

## Data Availability

All data analyzed in this study are publicly available from TCGA (https://portal.gdc.cancer.gov), ICGC (https://dcc.icgc.org), GTEx (https://gtexportal.org), TIMER2.0, GEPIA2, UALCAN, HPA, cBioPortal, SMART, Pathway Commons, Enrichr, STRING, miRDB, TargetScan, miRWalk, dbDEMC, and starBase. Raw experimental data generated in this study are available from the corresponding author upon reasonable request.

## Supplementary material

SUPPLEMENTARY FIGURE S1 Expression levels of EIF3M in various human organs and its cellular localization. **(A)** Expression levels of EIF3M across 50 organs in multiple human systems. **(B)** Schematic diagram of subcellular localization analysis for EIF3M expression in cells. **(C)** Immunofluorescence images of EIF3M in A-431, U2OS, and U-251MG cell lines. EIF3M, nucleus, and microtubules are labeled with green, blue, and red, respectively.

SUPPLEMENTARY FIGURE S2 Comparison of EIF3M gene expression activity scores across multiple tumor tissues versus normal tissues.

SUPPLEMENTARY FIGURE S3 The correlation between EIF3M expression and different histopathological tumor stages as well as patient survival. **(A)** The correlation between EIF3M expression and tumor stage in four statistically significant tumor tissues. The correlation between EIF3M expression and **(B)** Progression-Free Survival as well as Disease-Free Survival in cancer patients.

SUPPLEMENTARY FIGURE S4 Functional Characterization and Pathway Enrichment Analysis of EIF3M-Associated Genes and Proteins. **(A)** Genes correlated with EIF3M. **(B)** Pan-cancer expression profiling of EIF3M-associated genes. **(C)** Pathway enrichment and **(D)** ontological analysis of EIF3M-associated genes. **(E)** Proteins exhibiting close interaction relationships with EIF3M. **(F)** Gene Ontology Analysis of EIF3M-Interacting Proteins.

SUPPLEMENTARY FIGURE S5 Screening of EIF3M-targeting miRNAs. **(A)** Prediction of miRNA candidates through intersection analysis of miRDB, TargetScan, and miRWalk databases. **(B)** Meta-analysis heatmap of 41 EIF3M-targeting miRNAs across pan-cancer versus matched normal tissues.

SUPPLEMENTARY FIGURE S6
**(A)** Correlation between hsa-miR-199b-3p with EIF3M expression across various cancers.
