# Supplementary material for: EIF3M as a pan-cancer biomarker: prognostic significance and immune infiltration association
Source: Front Mol Biosci. 2025 Nov 18;12:1697083. doi: 10.3389/fmolb.2025.1697083 (PMC12669982; doi:10.3389/fmolb.2025.1697083)
Supplement: Supplementary file 1 [file Supplementaryfile2.zip › Supplementary Tables/Table S1.docx]

**Table S1 Small interfering RNA sequences for the target (EIF3M) genes are presented below.**

Table S1 Small interfering RNA sequences

| si-RNA | Sequence (5'-3') |
| --- | --- |
| si-NC | SS Sequence: UUCUCCGAACGUGUCACGUTT  AS Sequence: ACGUGACACGUUCGGAGAATT |
| si-EIF3M | SS Sequence: GAUGUUGAAGCAUUUGUUAUU  AS Sequence: UAACAAAUGCUUCAACAUCAU |
