# Supplementary material for: EIF3M as a pan-cancer biomarker: prognostic significance and immune infiltration association
Source: Front Mol Biosci. 2025 Nov 18;12:1697083. doi: 10.3389/fmolb.2025.1697083 (PMC12669982; doi:10.3389/fmolb.2025.1697083)
Supplement: Supplementary file 1 [file Supplementaryfile2.zip › Supplementary Tables/Table S10.docx]

**Table S10** Top five miRNA-target interactions in enrichmentanalysis of hsa-miR-199b-3p

| **pathwayName** | **log10(p-val)** | **log10(FDR)** |
| --- | --- | --- |
| **KEGG** |  |  |
| KEGG_Mapk_Signaling_Pathway | -18.92645 | -16.71426 |
| KEGG_Pathways_In_Cancer | -17.77964 | -15.86848 |
| KEGG_Focal_Adhesion | -17.2644 | -15.52934 |
| KEGG_Regulation_Of_Actin_Cytoskeleton | -16.42243 | -14.81231 |
| KEGG_Neurotrophin_Signaling_Pathway | -15.26997 | -13.75675 |
| **Disease Ontology** |  |  |
| Of_Gene-disease_Association | -41.53348 | -38.78607 |
| In_Tumors | -27.73465 | -25.28827 |
| In_Breast_Cancer | -26.93135 | -24.66106 |
| In_Prostate_Cancer | -19.16659 | -17.02123 |
| Of_Cancer | -17.2255 | -15.17706 |
| **Biological Processe** |  |  |
| GOBP_Positive_Regulation_Of_Nucleobase_Containing_Compound_Metabolic_Process | -76.63465 | -72.89516 |
| GOBP_Positive_Regulation_Of_Biosynthetic_Process | -75.26942 | -71.83096 |
| GOBP_Cellular_Macromolecule_Localization | -73.59791 | -70.33554 |
| GOBP_Regulation_Of_Intracellular_Signal_Transduction | -68.97741 | -65.83997 |
| GOBP_Regulation_Of_Protein_Modification_Process | -66.87656 | -63.83603 |
| **Molecular Function** |  |  |
| GOMF_Enzyme_Binding | -68.4878 | -65.45357 |
| GOMF_Transcription_Regulator_Activity | -55.59786 | -52.86466 |
| GOMF_Identical_Protein_Binding | -52.29043 | -49.73332 |
| GOMF_Rna_Binding | -49.7899 | -47.35773 |
| GOMF_Sequence_Specific_Dna_Binding | -47.83628 | -45.50103 |
