# Supplementary material for: EIF3M as a pan-cancer biomarker: prognostic significance and immune infiltration association
Source: Front Mol Biosci. 2025 Nov 18;12:1697083. doi: 10.3389/fmolb.2025.1697083 (PMC12669982; doi:10.3389/fmolb.2025.1697083)
Supplement: Supplementary file 1 [file Supplementaryfile2.zip › Supplementary Tables/Table S11.docx]

**Table S11** The stromal and immune scores for each cancer type are presented in the results

| **CancerType** | **StromalScore** | **ImmuneScore** |
| --- | --- | --- |
| ACC | **0.00057** | **0.00047** |
| BRCA | **3.00E-08** | 0.92846 |
| CHOL | 0.16578 | **0.02333** |
| COAD | **1.44E-09** | **1.97E-11** |
| DLBC | 0.54627 | **0.00264** |
| GBM | **0.01558** | 0.10029 |
| HNSC | **6.36e-11** | 0.05699 |
| LAML | **0.00267** | **0.00269** |
| LGG | **1.14e-07** | **0.03252** |
| LIHC | **0.00209** | 0.71856 |
| LUAD | **0.00182** | 0.21315 |
| OV | **1.45e-07** | **0.02574** |
| PAAD | **0.00092** | 0.34134 |
| PRAD | **0.00306** | 0.66937 |
| READ | **0.01731** | **0.00915** |
| SARC | **0.00196** | 0.87416 |
| SKCM | **4.23e-05** | **0.00361** |
| STAD | **0.00023** | 0.96277 |
| THCA | **1.09e-06** | **1.91e-07** |
| UCEC | **0.00317** | 0.17849 |
| UVM | **8.76e-06** | **1.74e-06** |
