# Supplementary material for: EIF3M as a pan-cancer biomarker: prognostic significance and immune infiltration association
Source: Front Mol Biosci. 2025 Nov 18;12:1697083. doi: 10.3389/fmolb.2025.1697083 (PMC12669982; doi:10.3389/fmolb.2025.1697083)
Supplement: Supplementary file 1 [file Supplementaryfile2.zip › Supplementary Tables/Table S2.docx]

**Table S2 The RT qPCR reaction system is presented below.**

Table S2-1 Reaction System Configuration

| 2xNovoScripPlus Stand cDNA, Synthesis.SuperMix | 10ul |
| --- | --- |
| Template RNA | Total RNA(0.1ng -1µg) |
|  | mRNA(≥10pg) |
|  | Specific RNA（≥0.01pg） |
| gDNA Purge | 1ul |
| RNase Free Water | Up to 20µl |

Table S2-2 Reaction Program

| Temperature | Time |
| --- | --- |
| 50 | 15min |
| 85 | 5s |
