# Supplementary material for: EIF3M as a pan-cancer biomarker: prognostic significance and immune infiltration association
Source: Front Mol Biosci. 2025 Nov 18;12:1697083. doi: 10.3389/fmolb.2025.1697083 (PMC12669982; doi:10.3389/fmolb.2025.1697083)
Supplement: Supplementary file 1 [file Supplementaryfile2.zip › Supplementary Tables/Table S3.docx]

**Table S3 Primer sequences for the reference (GAPDH) and target (EIF3M) genes are presented below.**

Table S3 EIF3M Primer Sequence

| EIF3M | Sequence (5'-3') |
| --- | --- |
| EIF3M-forward primer sequence | CGGCCTTCATCGACATCAGT |
| EIF3M-reverse primer sequence | AGTCCACCTTCCGAGTTCTCT |
| GAPDH-forward primer sequence | CAAGGTCATCCATGACAACTTTG |
| GAPDH-reverse primer sequence | GTCCACCACCCTGTTGCTGTAG |
