# Supplementary material for: EIF3M as a pan-cancer biomarker: prognostic significance and immune infiltration association
Source: Front Mol Biosci. 2025 Nov 18;12:1697083. doi: 10.3389/fmolb.2025.1697083 (PMC12669982; doi:10.3389/fmolb.2025.1697083)
Supplement: Supplementary file 1 [file Supplementaryfile2.zip › Supplementary Tables/Table S4.docx]

**Table S4 The immunohistochemistry sample information sourced from the HPA database.**

| **Normal** | **Cancer** |
| --- | --- |
| **Lung**  **HPA031063** Female, age 49 Lung (T-28000) Bronchus (T-26000) Normal tissue, NOS (M-00100) Patient id: 2268 | **Lung cancer**  **HPA031063** Male, age 49 Lung (T-28000) Adenocarcinoma, NOS (M-81403) Patient id: 3003 |
| **Liver**  **HPA031063** Female, age 63 Liver (T-56000) Normal tissue, NOS (M-00100) Patient id: 3222 | **Liver cancer**  **HPA031063** Female, age 73 Liver (T-56000) Carcinoma, Hepatocellular, NOS (M-81703) Patient id: 2766 |
| **Thyroid gland**  **HPA031063** Female, age 22 Thyroid gland (T-96000) Normal tissue, NOS (M-00100) Patient id: 1712 | **Thyroid cancer**  **HPA031063** Male, age 33 Thyroid gland (T-96000) Papillary adenocarcinoma, NOS (M-82603) Patient id: 3267 |
| **Pancreas**  **HPA031063** Female, age 35 Pancreas (T-59000) Carcinoid, malignant, NOS (M-82403) Normal tissue, NOS (M-00100) Patient id: 2032 | **Pancreatic cancer**  **HPA031063** Female, age 56 Pancreas (T-59000) Adenocarcinoma, NOS (M-81403) Patient id: 3233 |
| **Endometrium 1**  **HPA031063** Female, age 33 Endometrium (T-84000) Normal tissue, NOS (M-00100) Patient id: 2941 | **Endometrial cancer**  **HPA031063** Female, age 70 Uterus (T-82000) Endometrium (T-84000) Adenocarcinoma, NOS (M-81403) Patient id: 2118 |
