# Supplementary material for: EIF3M as a pan-cancer biomarker: prognostic significance and immune infiltration association
Source: Front Mol Biosci. 2025 Nov 18;12:1697083. doi: 10.3389/fmolb.2025.1697083 (PMC12669982; doi:10.3389/fmolb.2025.1697083)
Supplement: Supplementary file 1 [file Supplementaryfile2.zip › Supplementary Tables/Table S5.docx]

**Table S5** The grouping and sample information used for each cancer type in the gene activity score analysis

| **CancerType** | **Tumor** | **Normal** | **CancerType** | **Tumor** | **Normal** |
| --- | --- | --- | --- | --- | --- |
| ACC | 79 | 0 | LUAD | 526 | 59 |
| BLCA | 411 | 19 | MESO | 86 | 0 |
| BRCA | 1104 | 113 | OV | 379 | 0 |
| CESC | 306 | 3 | PAAD | 178 | 4 |
| CHOL | 36 | 9 | PCPG | 183 | 3 |
| COAD | 471 | 41 | PRAD | 499 | 52 |
| DLBC | 48 | 0 | READ | 167 | 10 |
| ESCA | 162 | 11 | SARC | 263 | 2 |
| GBM | 168 | 5 | SKCM | 471 | 1 |
| HNSC | 502 | 44 | TGCT | 156 | 0 |
| KICH | 65 | 24 | THCA | 510 | 58 |
| KIRC | 535 | 72 | THYM | 119 | 2 |
| KIRP | 289 | 32 | UCEC | 548 | 35 |
| LAML | 151 | 0 | UCS | 56 | 0 |
| LGG | 529 | 0 | UVM | 80 | 0 |
| LIHC | 374 | 50 |  |  |  |
