# Supplementary material for: EIF3M as a pan-cancer biomarker: prognostic significance and immune infiltration association
Source: Front Mol Biosci. 2025 Nov 18;12:1697083. doi: 10.3389/fmolb.2025.1697083 (PMC12669982; doi:10.3389/fmolb.2025.1697083)
Supplement: Supplementary file 1 [file Supplementaryfile2.zip › Supplementary Tables/Table S8.docx]

**Table S8** Grouping and sample information used for various cancers in CpG-dense regions methylation analysis

| **Tissue** | **Tumor** | **Normal** | **Tissue** | **Tumor** | **Normal** |
| --- | --- | --- | --- | --- | --- |
| ACC | 79 | 0 | LUSC | 364 | 41 |
| BLCA | 413 | 21 | MESO | 87 | 0 |
| BRCA | 783 | 87 | OV | 10 | 0 |
| CESC | 308 | 3 | PAAD | 184 | 10 |
| CHOL | 36 | 9 | PCPG | 183 | 3 |
| COAD | 288 | 34 | PRAD | 496 | 50 |
| DLBC | 48 | 0 | READ | 93 | 7 |
| ESCA | 184 | 15 | SARC | 261 | 4 |
| GBM | 150 | 1 | SKCM | 473 | 2 |
| HNSC | 525 | 50 | STAD | 393 | 2 |
| KICH | 65 | 0 | TGCT | 138 | 0 |
| KIRC | 313 | 157 | THCA | 511 | 56 |
| KIRP | 272 | 43 | THYM | 124 | 2 |
| LAML | 194 | 0 | UCEC | 419 | 33 |
| LGG | 527 | 0 | UCS | 57 | 0 |
| LIHC | 376 | 50 | UVM | 80 | 0 |
| LUAD | 458 | 30 |  |  |  |
