# Supplementary material for: EIF3M as a pan-cancer biomarker: prognostic significance and immune infiltration association
Source: Front Mol Biosci. 2025 Nov 18;12:1697083. doi: 10.3389/fmolb.2025.1697083 (PMC12669982; doi:10.3389/fmolb.2025.1697083)
Supplement: Supplementary file 1 [file Supplementaryfile2.zip › Supplementary Tables/Table S9.docx]

**Table S9** Grouping and sample information used for various cancers in promoter methylation analysis

| **Tissue** | **Tumor** | **Normal** | **Tissue** | **Tumor** | **Normal** |
| --- | --- | --- | --- | --- | --- |
| BLCA | 418 | 21 | LUAD | 473 | 32 |
| COAD | 37 | 313 | LUSC | 370 | 42 |
| ESCA | 185 | 16 | PAAD | 184 | 10 |
| HNSC | 528 | 50 | PRAD | 502 | 50 |
| KIRP | 275 | 45 | TGCT | 69 | 63 |
| LIHC | 377 | 50 | UCEC | 438 | 46 |
