# Supplementary figures and images for: EIF3M as a pan-cancer biomarker: prognostic significance and immune infiltration association
Source: Front Mol Biosci. 2025 Nov 18;12:1697083. doi: 10.3389/fmolb.2025.1697083 (PMC12669982; doi:10.3389/fmolb.2025.1697083)

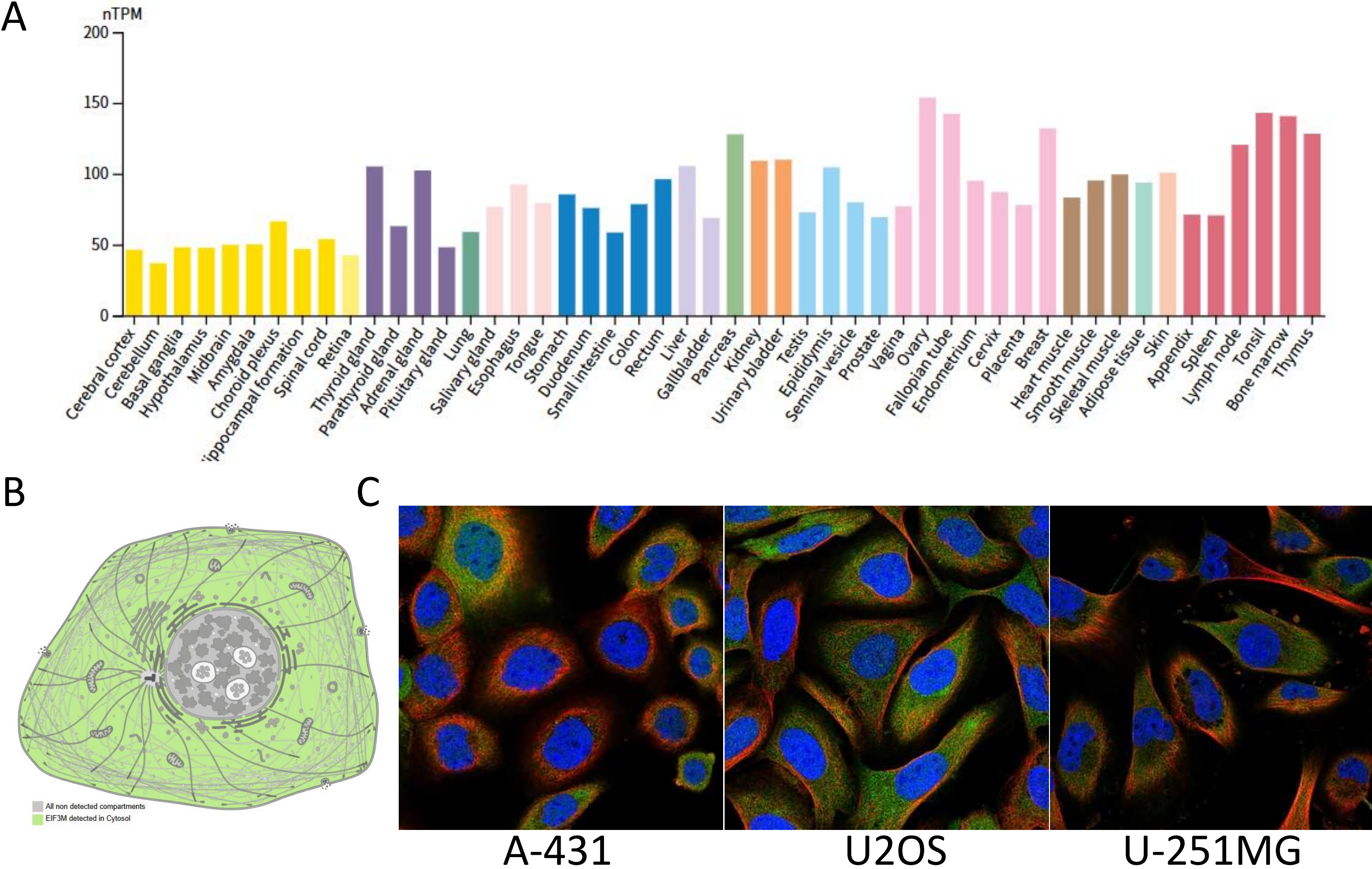

Supplement: Supplementary file 2 [file Supplementaryfile1.zip › Supplementary Figures/Supplementary Figure 1.tif]

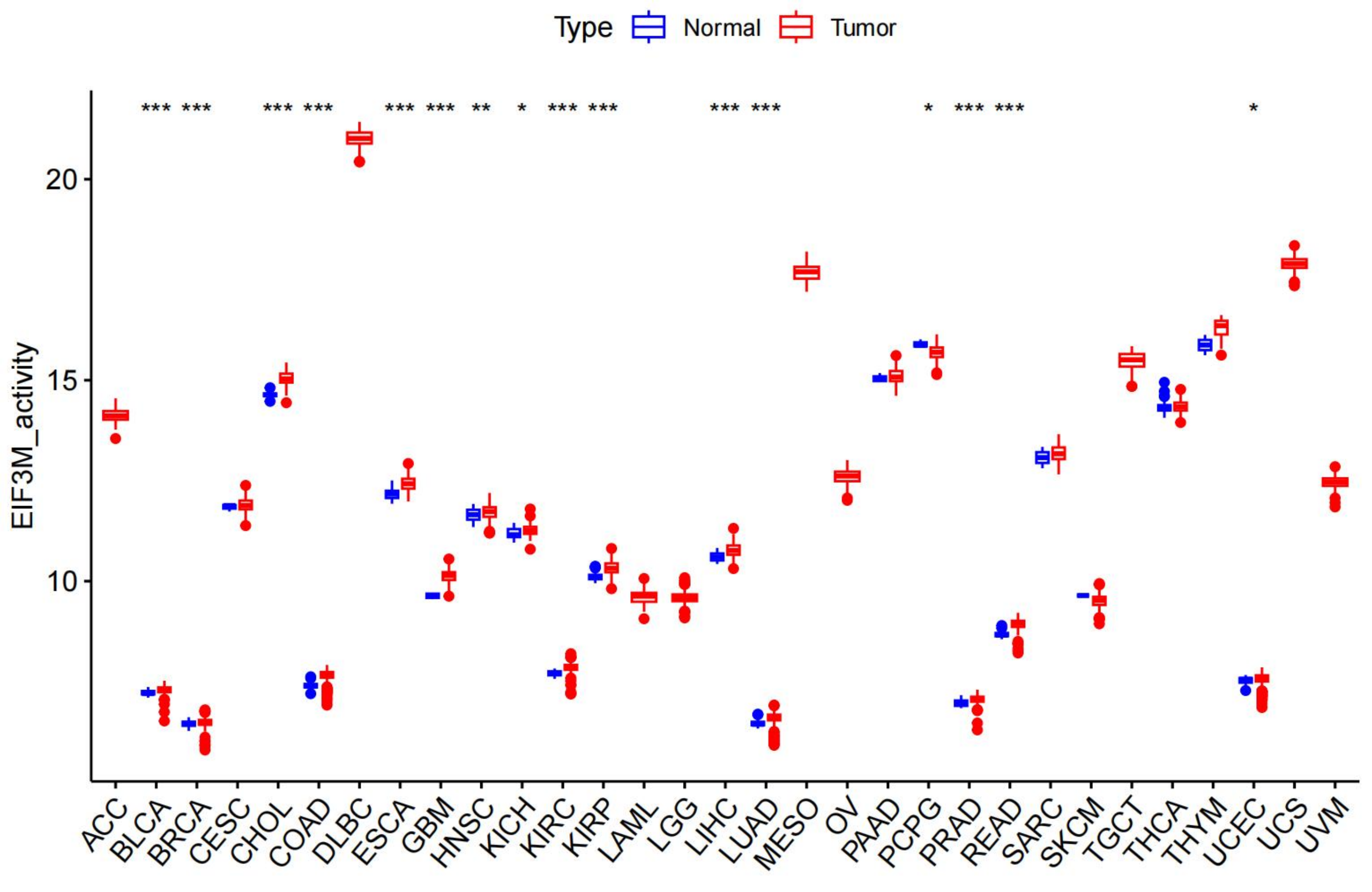

Supplement: Supplementary file 2 [file Supplementaryfile1.zip › Supplementary Figures/Supplementary Figure 2.tif]

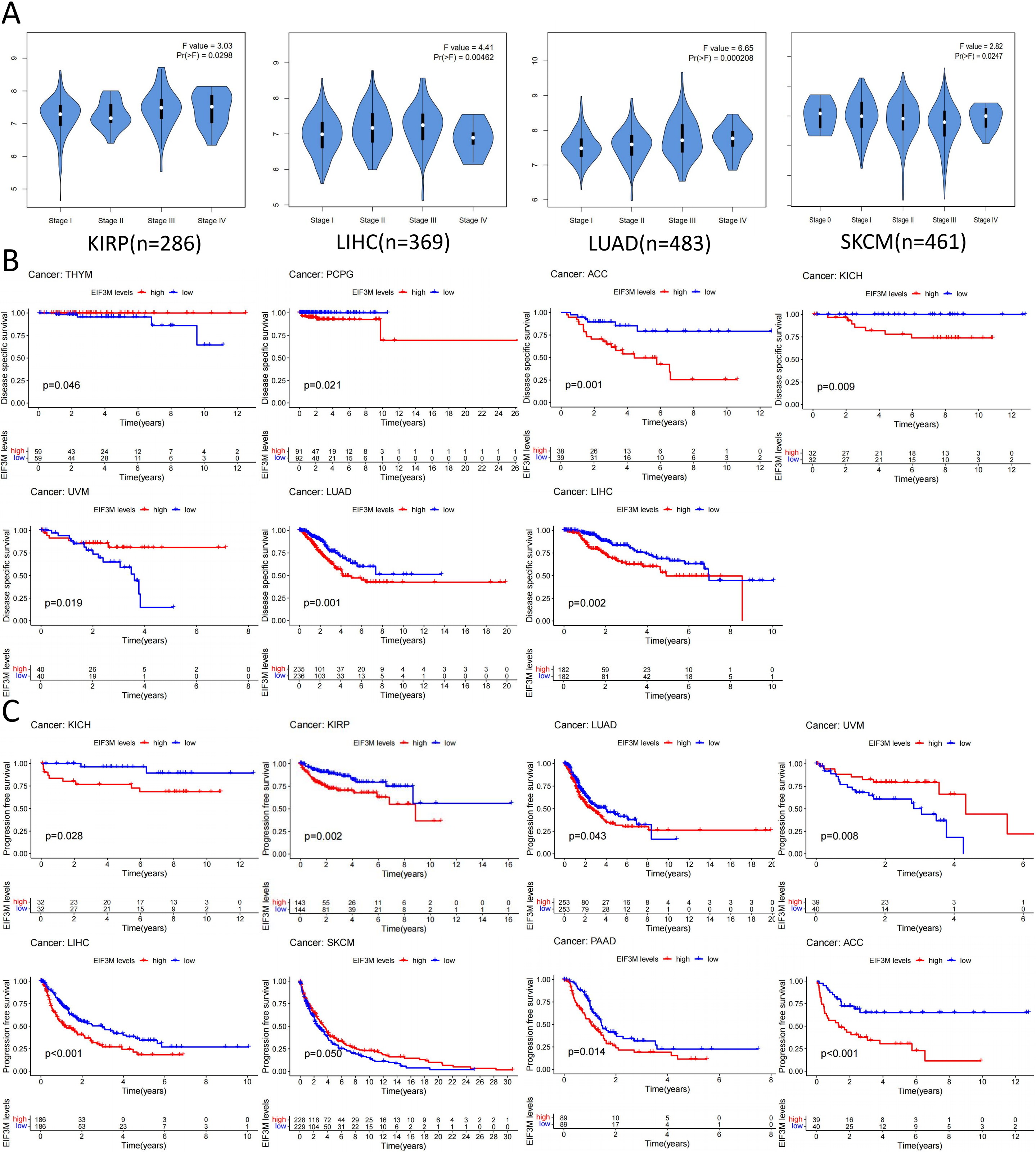

Supplement: Supplementary file 2 [file Supplementaryfile1.zip › Supplementary Figures/Supplementary Figure 3.tif]

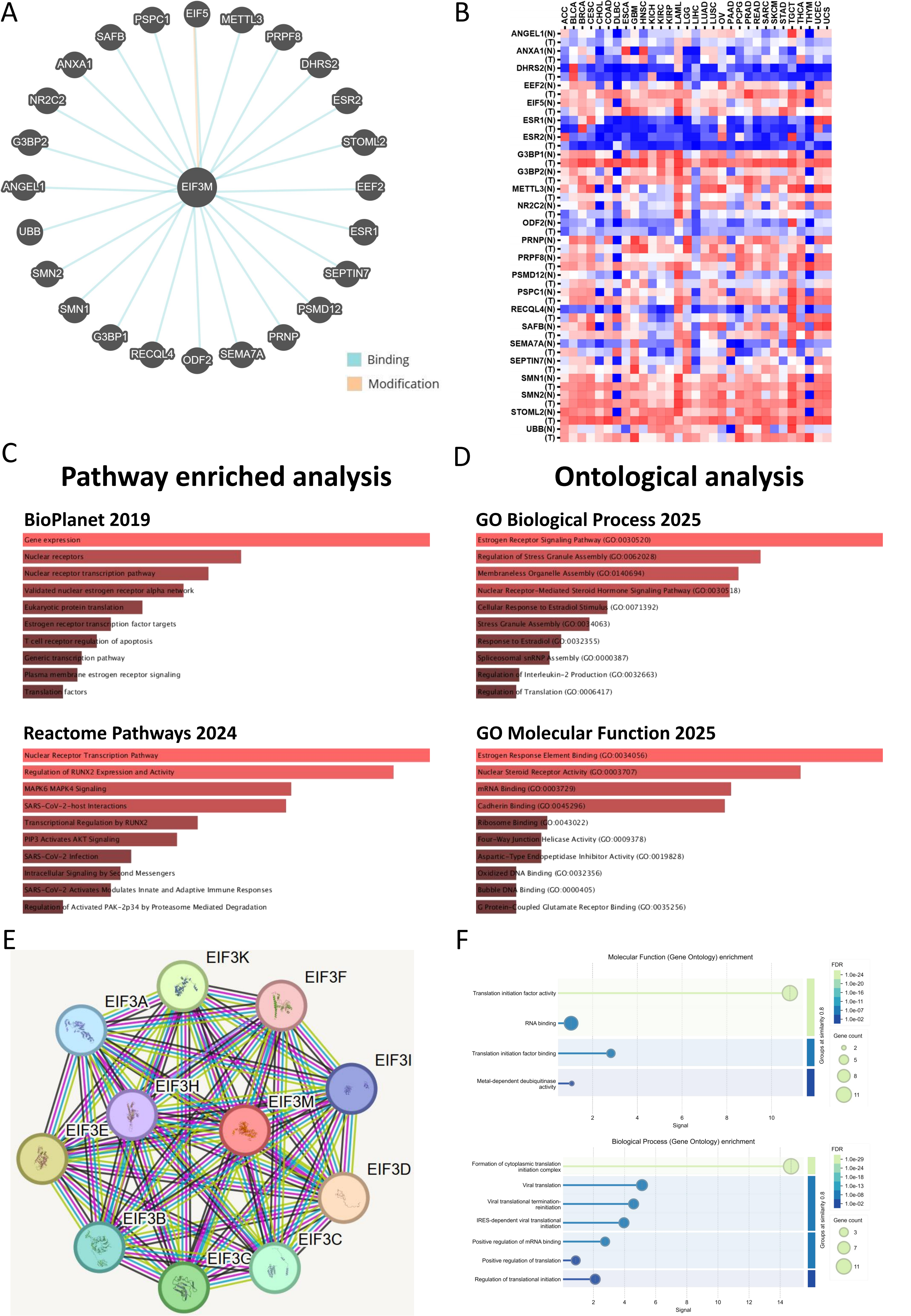

Supplement: Supplementary file 2 [file Supplementaryfile1.zip › Supplementary Figures/Supplementary Figure 4.tif]

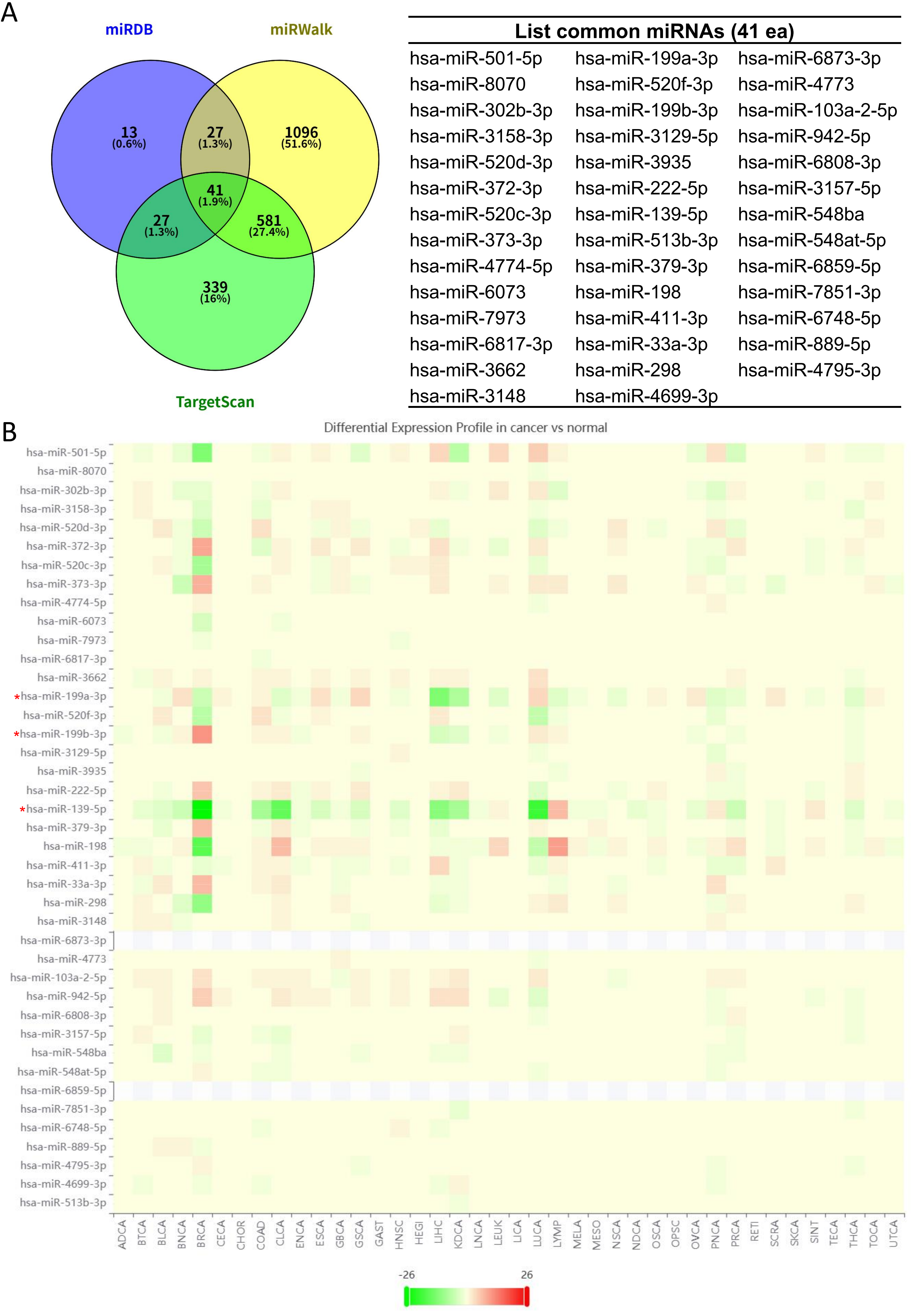

Supplement: Supplementary file 2 [file Supplementaryfile1.zip › Supplementary Figures/Supplementary Figure 5.tif]

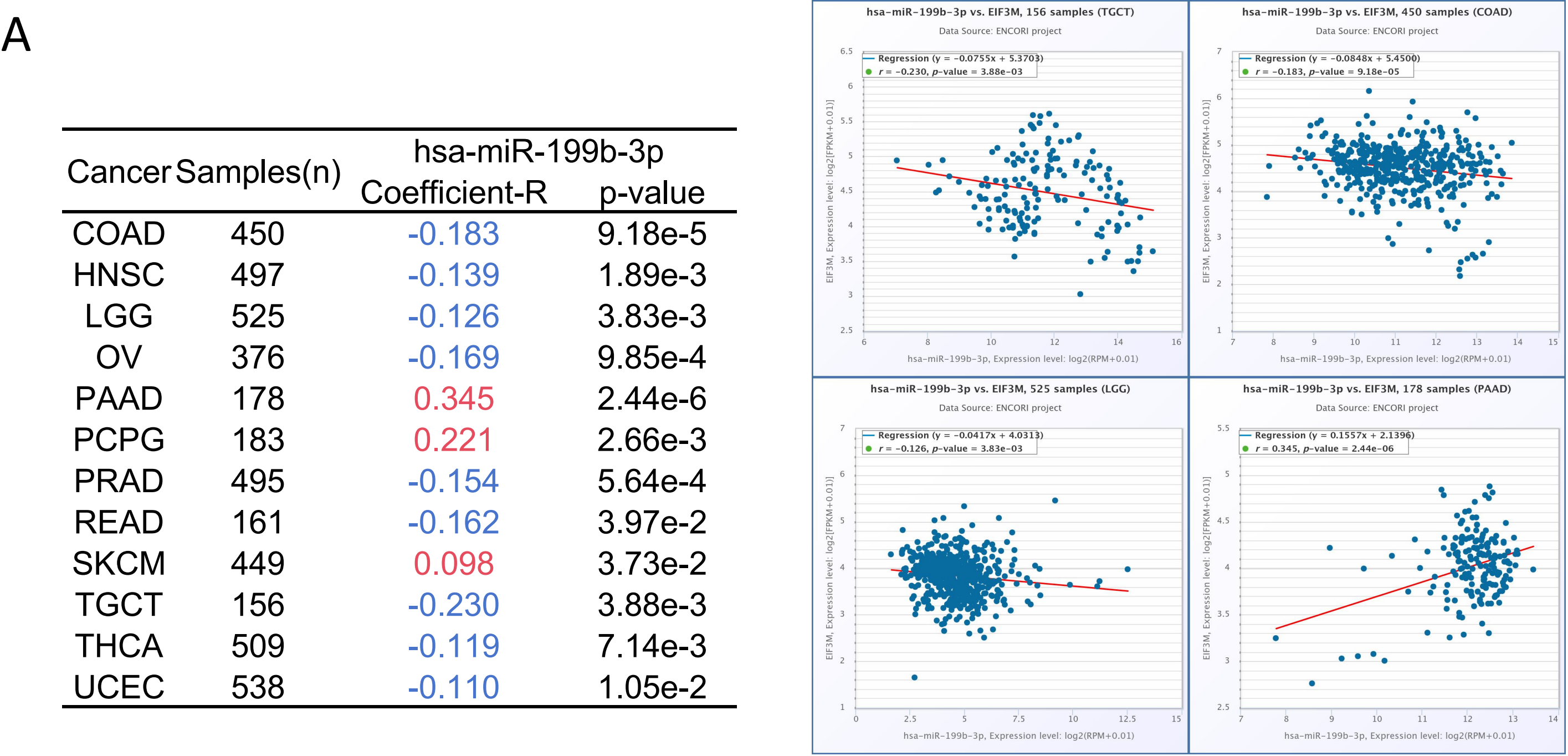

Supplement: Supplementary file 2 [file Supplementaryfile1.zip › Supplementary Figures/Supplementary Figure 6.tif]
